# Bridging the birth dose gap: A scoping review of implementation challenges for birth dose and rotavirus vaccines in low- and middle-income countries

**DOI:** 10.1371/journal.pgph.0006910

**Published:** 2026-11-06

**Authors:** Mei Neni Sitaresmi, Risalia Reni Arisanti, Vicka Oktaria, Adzilla Fikria, Pramitha Esha Nirmala Dewi, Alya Nur Fadhilah, Indah Kartika Murni, Indah Rosmawati, Intan Cahyani, Candora Tambunan, Justin C. Graves, Navpreet Singh, Stella Benita, Niken Widyastuti, Tetrawindu Hidayatullah, Atiek Anartati, Endang Budi Hastuti, Devi Anisiska, Prima Yosephine

**Affiliations:** 1 Centre for Child Health – Pediatric Research Office, Faculty of Medicine, Public Health and Nursing, Universitas Gadjah Mada, Yogyakarta, Indonesia; 2 Child Health Department, Faculty of Medicine, Public Health and Nursing, Universitas Gadjah Mada, Yogyakarta, Indonesia; 3 Department of Biostatistics, Epidemiology, and Population Health, Faculty of Medicine, Public Health and Nursing, Universitas Gadjah Mada, Yogyakarta, Indonesia; 4 Department of Pharmacy Profession, Universitas Muhammadiyah Yogyakarta, Yogyakarta, Indonesia; 5 Clinton Health Access Initiative, Indonesia Office, Jakarta, Indonesia; 6 Clinton Health Access Initiative, Analytics and Implementation Research Team, Boston, Massachusetts, United States of America; 7 Clinton Health Access Initiative, Global Vaccine Delivery, Boston, Massachusetts, United States of America; 8 Ministry of Health, Republic of Indonesia, Expanded Programme on Immunization, Jakarta, Indonesia; Christian Medical College Vellore, INDIA

## Abstract

Rotavirus vaccination (RVV) has substantially reduced childhood diarrhoeal morbidity and mortality globally. However, interest in neonatal or birth-dose schedules is increasing to provide earlier protection and potentially reduce intussusception risk. Understanding barriers and implementation challenges of current RVV and other birth-dose vaccines (BDVs), including hepatitis B birth dose, Bacillus Calmette–Guérin, and oral polio vaccine, is essential to inform this transition in low- and middle-income countries. This review therefore aims to examine the supply- and demand-side factors influencing the uptake, timeliness, and implementation of RVV and BDV in low- and middle-income countries. A scoping review was conducted across PubMed, Scopus, Cochrane, and grey literature from Portal Garuda from database inception to 5 March 2025. Original studies assessing determinants, enablers, and barriers of any BDV or RVV implementation, uptake, or timeliness were included. An analytical framework was developed inductively to classify factors as supply- or demand-side and was refined through discussions among reviewers. From 8,502 identified records, a total of 93 articles were included in the review. Identified supply-side enablers and barriers encompassed domains related to policy and governance, health system capacity, healthcare accessibility, and programmatic interventions. On the demand side, key enablers included higher caregiver education, higher socioeconomic status, health facility birth, and older maternal age, whereas major barriers included home birth, lower socioeconomic status, rural residence for BDV, and incomplete or delayed uptake of other routine vaccinations for RVV. Implementation and uptake of BDV and RVV were influenced by both supply- and demand-side factors, including policy support, health system capacity and access, targeted interventions, and socioeconomic disparities in vaccine uptake. Transitioning from infant-dose to birth-dose RVV will require addressing existing implementation challenges and ensuring timely vaccination at birth to achieve equitable coverage.

## Introduction

Rotavirus (RV) is a major cause of diarrhoeal diseases in children younger than five years, responsible for an estimated 120,000 deaths and over 10.4 million DALYs in 2021, with the burden particularly higher in the age group of 0–2 years [1]. RV vaccination (RVV) has been effective in reducing acute gastroenteritis across various settings, with effectiveness ranging from 54% to 94% in different mortality strata and country settings [2–7], following its worldwide introduction in different timeframes. However, countries’ performance remains variable; WHO/UNICEF Estimates of National Immunization Coverage recorded RVV coverage at 59% in 2024, ranging from 2% in Iran to 99% in Uzbekistan [8].

A recent development is the emergence of an RV birth dose vaccine, which has not yet been prequalified by WHO but shows potential to establish early protection [9–14]. The birth dose vaccine, RV3-BB, followed a three-dose schedule at the first 0–5 days of life, 8–10 weeks and 14–16 weeks [9,10,13]. Full RV3-BB doses achieved 94% efficacy against severe rotavirus gastroenteritis at 12 months of age and 75% efficacy at 18 months in a phase 2b clinical trial, with no cases of intussusception observed in the neonatal-schedule cohort, comparable to other WHO-prequalified RVV available [7,9].

Vaccination at birth also offers programmatic advantages, with coverage and timeliness often higher than for vaccines administered later in infancy [15,16]. For instance, a study in the United States found that HepB-BD vaccination at birth is associated with higher completion of subsequent vaccination series [17]. However, inequalities were evident, where worldwide birth dose vaccinations’ (BDVs) coverage was recorded at 88% for the Bacillus Calmette–Guérin (BCG) and 45% for the Hepatitis B birth dose (HepB-BD), variable between settings [18,19]. Coverage was found to be positively correlated with institutional delivery rates, skilled birth attendant rates, adult health literacy, total health expenditure per capita, and negatively correlated with number of live births [20].

These patterns reflect broader and persistent inequalities in childhood immunisation. While high-income countries record high coverage of childhood vaccination, coverage is suboptimal in several regions, most pronounced in Sub-Saharan Africa [21]. During the COVID-19 period, inequality grew even larger due to higher coverage decline, increasing numbers of zero-dose children, and a lag in childhood vaccination recovery in low- and middle-income countries after pandemics [22]. Global analysis (2015–2022) also found that despite high vaccine confidence observed in low-income and middle-income countries, coverage lagged compared with high-income countries [23]. This disconnect suggests that barriers to vaccination extend beyond individual-level factors, highlighting the critical role of health system constraints and broader structural determinants.

Previous reviews have highlighted the complex interplay between supply- and demand-side factors influencing childhood vaccination in LMICs [24,25], but limited evidence has specifically synthesised determinants of RVV and BDV implementation within this context. This gap is increasingly relevant given recent developments in RVV, including candidates designed for administration at birth, which may offer new opportunities to improve early-life protection but also introduce additional implementation challenges. Understanding the determinants of the uptake and timeliness of BDV and RVV is essential to identify implementation patterns and possible strategies to improve coverage and equitable access, particularly for the planned switch to RV birth dose vaccination. Therefore, this review aims to explore enablers and barriers of RVV and BDV in low- and middle-income countries, both from the supply and demand sides.

## Methods

### Search strategy

This scoping review was conducted according to the Preferred Reporting Items for Systematic Reviews and Meta-Analyses (PRISMA) guideline. Searching was conducted from 13 February 2025–5 March 2026 at PubMed, Scopus, and Cochrane to find relevant peer-reviewed literature and at Portal Garuda, an Indonesian database of grey literature, with no restriction of publication year. The complete keyword for literature searching is presented in Table 1 in S1 Appendix. The scoping review protocol is available at Open Science Framework (https://doi.org/10.17605/OSF.IO/E6H49) [26].

### Eligibility criteria

Studies were included if they met the following criteria: (i) assessed determinants, enablers, and barriers of any BDV or RVV implementation, uptake, or timeliness in low- and middle-income countries; (ii) were published in the English or Indonesian language; and (iii) were original research. Studies were eligible if they assessed BDVs or RVV either as a primary objective or as part of a subgroup analysis. No restrictions were placed on study period or design; therefore, quantitative, qualitative, and mixed-method studies from any year were included. Studies for which the full text could not be obtained were excluded from the review.

We included only original research to directly summarise the determinants reported in the original studies and to allow the context in which these determinants were identified to be considered. Reviews were excluded to avoid duplication of enablers and barriers reported in the underlying original research. Accordingly, non-original research, including study protocols, literature reviews, systematic reviews, meta-analyses, abstract conferences, or reports, was excluded. Studies published in non-English or non-Indonesian languages or for which full text was not found or unavailable to be accessed were also excluded from the review.

### Study selection

The results from the search were organised, and duplicate references were eliminated using Rayyan [27]. Four reviewers (AF, RRA, PEND, ANF) independently screened all titles and abstracts identified using the inclusion and exclusion criteria for suitability. Full-text articles for research judged relevant were retrieved, and two reviewers (AF and ANF) then assessed the publications’ eligibility. Any discrepancies were discussed and solved by consensus among four reviewers (AF, RRA, PEND, ANF).

### Data extraction and synthesis

A data extraction spreadsheet was developed to capture methodological characteristics, including study setting (country, continent, World Bank income group classification in 2025, and sampling level), study period, design, study population, sample size, and data collection methods, as well as identified determinants, enablers or barriers and their significance. Information from the articles was retrieved using the same predefined form.

Determinants, enablers, and barriers of BDV and RVV uptake, timeliness, or implementation were categorised as either supply- or demand-side factors. Enablers were defined as variables identified as facilitating immunisation implementation, including programme delivery, improved coverage, or timeliness, whereas barriers were defined as factors hindering these outcomes. Determinants identified in quantitative studies were classified according to their association with immunisation outcomes, including coverage and timeliness, with positive associations categorised as enablers and negative associations as barriers. Factors related to service provision, ranging from policy-level determinants to facility capacity and available provider resources, were classified under the supply-side domain. In contrast, factors concerning service recipients, including sociodemographic characteristics, behaviours, perceptions, and healthcare decision-making processes, were classified under the demand-side domain.

An analytical framework classifying both supply- and demand-side factors was inductively developed from the findings and iteratively refined. Factors with similar characteristics were grouped under the same category. Findings were synthesised through discussions among reviewers, and the final results were presented after consensus was reached.

## Results

### Study characteristics

A total of 7,945 peer-reviewed articles and 557 grey literature records were identified from the databases. After removing duplicates (n = 3,098), 5,454 records underwent title and abstract screening, of which 596 peer-reviewed articles and 40 grey literature records were selected for full-text review. At the final stage, studies were excluded due to ineligible populations (n = 4) or outcomes (n = 417), wrong publication types (n = 18), unavailable full texts (n = 75), unresolved duplication (n = 1), or publication in languages other than English or Bahasa Indonesia (n = 23). In total, 93 articles were included in the review. A detailed flow chart of the study selection is presented in Fig 1.

**Fig 1:**
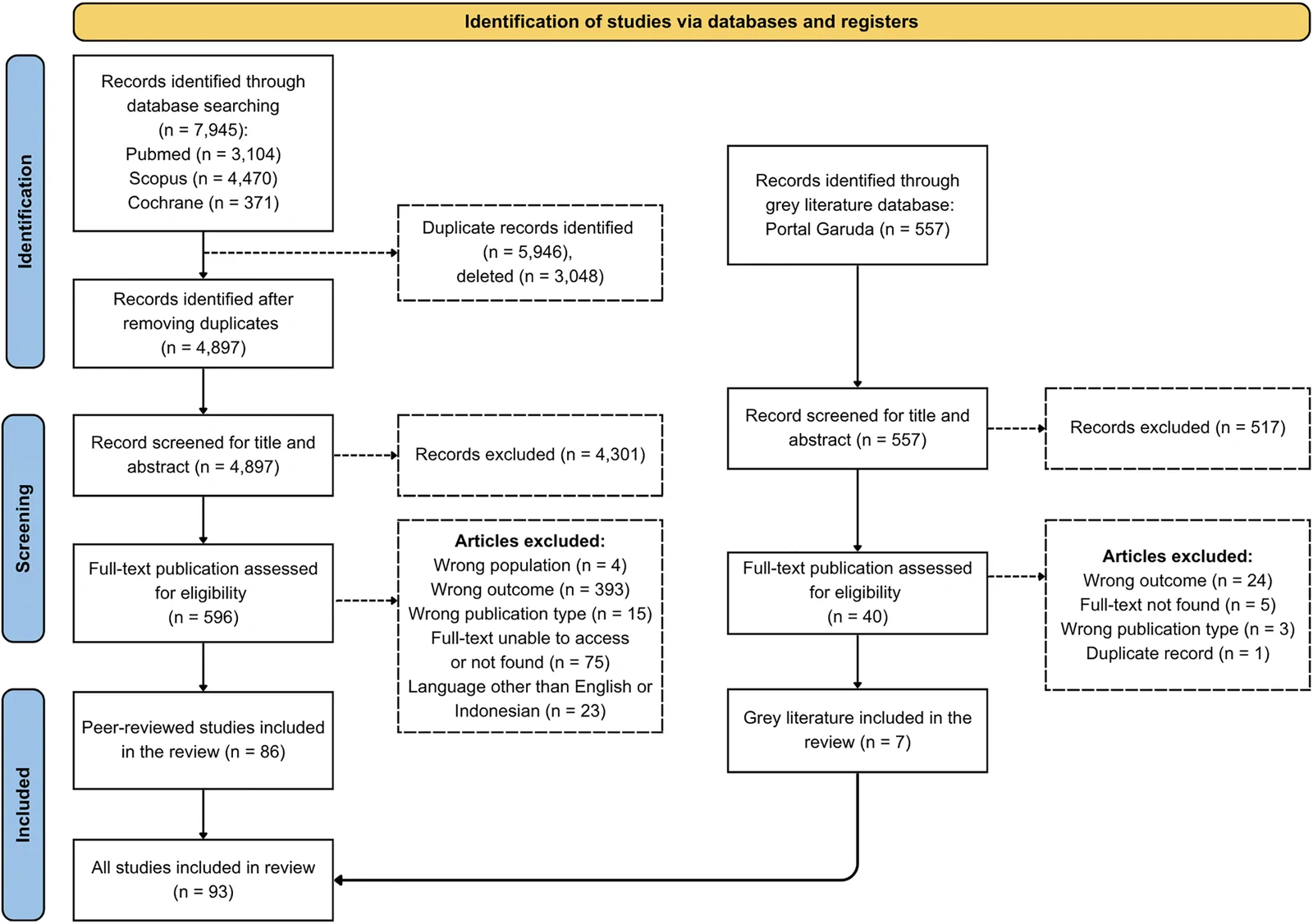
Screening and selection of literatures (PRISMA flow).

Among the included studies, 73 (78.5%) examined determinants of BDV and 24 (25.8%) assessed RVV. Demand-side factors were identified in most studies (n = 88, 94.6%), with enablers reported in 78 studies and barriers in 61 studies. Most studies were quantitative (n = 81, 87.1%) and used a cross-sectional design (n = 69, 74.2%). The primary data collection periods spanned from 1993 to 2025.

The geographic distribution of included studies was illustrated in Fig 2. Most were conducted in a single country (n = 89, 95.7%), primarily in Asia (n = 43, 46.2%) and Africa (n = 41, 44.1%). Based on the 2025 World Bank income classification, over half originated from lower-middle-income countries (n = 50, 53.8%), followed by upper-middle-income (n = 24) and low-income countries (n = 19). Data were mainly derived from subnational and national levels (each n = 40, 43.0%), with four studies using multinational data. Detailed study characteristics are presented in Tables 2–5 in S1 Appendix.

**Fig 2:**
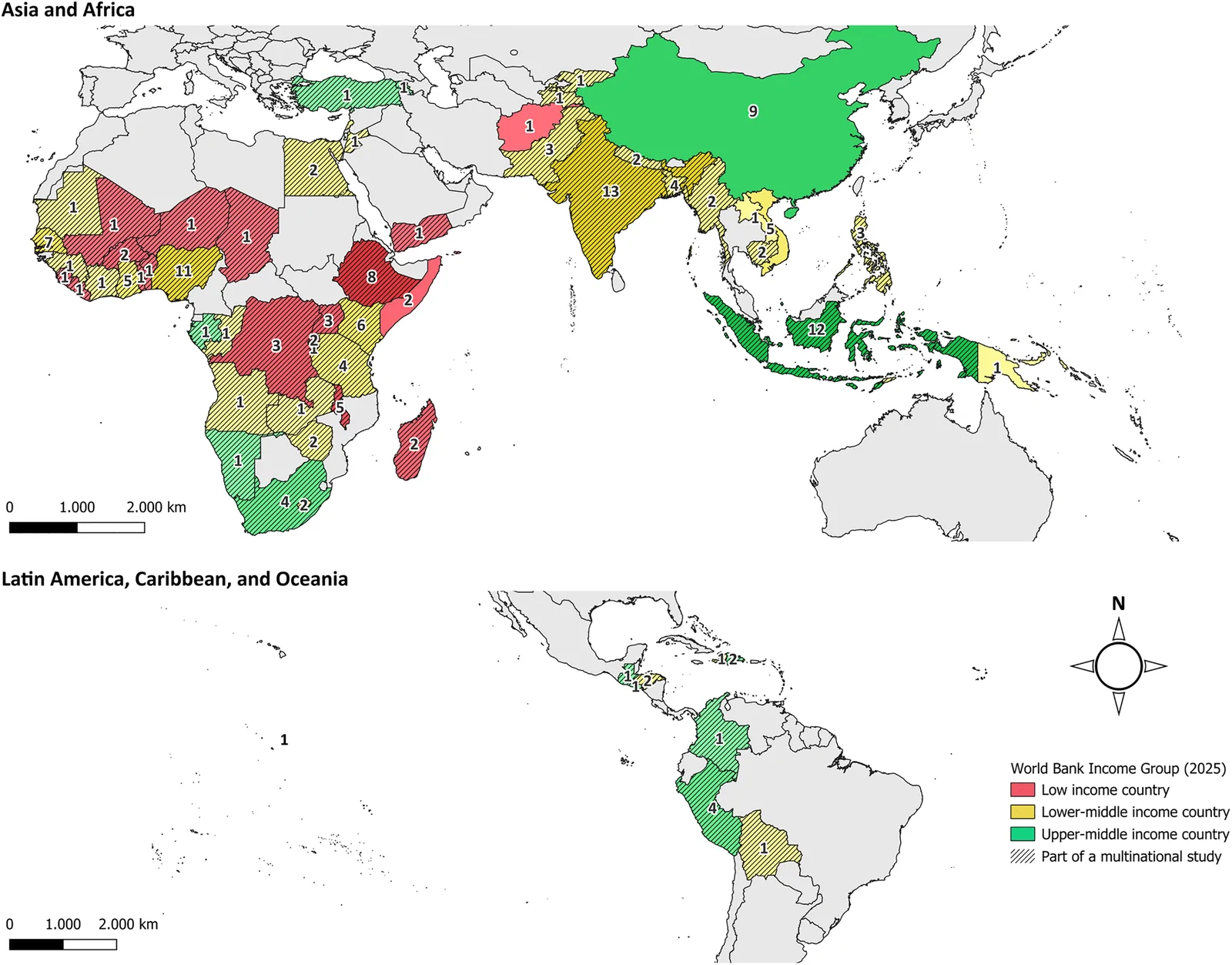
Geographic distribution of included studies by country and World Bank income group (2025). Labels indicate the number of studies conducted in each country. Map boundaries and geographic data were sourced from Natural Earth (https://www.naturalearthdata.com/).

### Description of framework

We categorised supply-side factors and demand-side factors into several overarching themes, as illustrated and defined in Table 1.

**Table 1:** Overarching themes in supply- and demand-side determinants.

Themes	Definition
***Supply-side factors***	
1. Policy and governance	Factors related to policies, planning, and management of immunisation programmes at the national level.
2. Health system capacity	The ability of the health system to deliver immunisation programmes, including the availability and adequacy of resources and facility-level management.
a. Facility readiness	The availability and adequacy of physical infrastructure and facilities for immunisation service delivery.
b. Health workforce	Factors related to immunisation providers, including workforce availability, competency, quality, and capacity-building efforts.
c. Vaccine logistics and products	Factors related to the availability and characteristics of vaccine products and immunisation logistics.
d. Monitoring and supervision	Factors associated with the monitoring and supervision of immunisation programme implementation at the facility level.
3. Health system accessibility	Aspects related to efforts by the health system to ensure accessibility of immunisation programmes, including service schedules, vaccination costs, and other access-related arrangements.
4. Programmatic intervention	Specific interventions implemented by the health system to improve immunisation programme delivery beyond the three domains above.
***Demand-side factors***	
1. Caregiver-related factor	Any factor associated with the child’s primary caregiver, including sociodemographic characteristics, behaviours, and decision-making processes regarding childhood immunisation.
a. Sociodemographic characteristics	Sociodemographic background of the primary caregiver.
b. Health service utilisation	Factors related to the primary caregiver’s use of and access to healthcare services.
c. Knowledge and perception of VPD and vaccination	Factors related to the primary caregiver’s knowledge and perceptions regarding vaccine-preventable diseases (VPDs), vaccination, healthcare services in general, and exposure to health-related information.
d. Social and cultural influence	Social and cultural factors influencing the primary caregiver’s health-related decisions or behaviours, originating from family, community, or broader social environments.
2. Child-related factor	Any factor specifically related to the child, independent of the primary caregiver, such as biological characteristics, health status, and other child-specific conditions.

Supply-side enablers and barriers were grouped into four themes: (1) policy and governance; (2) health system capacity; (3) health system accessibility; and (4) programmatic interventions. Identified demand-side determinants were classified as caregiver- or child-related factors. Caregiver-related factors were further grouped into sociodemographic characteristics, health service utilisation, knowledge and perceptions of vaccine-preventable diseases (VPDs) and vaccination, and social and cultural influences. Some determinants exhibited mixed associations with BDV or RVV uptake, being identified as enablers in certain studies and barriers in others. Therefore, these determinants were grouped under the category of mixed-direction determinants.

### Supply-side enablers and barriers

Supply-side enablers and barriers of BDV and RVV implementation are summarised in Figs 3–5.

**Fig 3:**
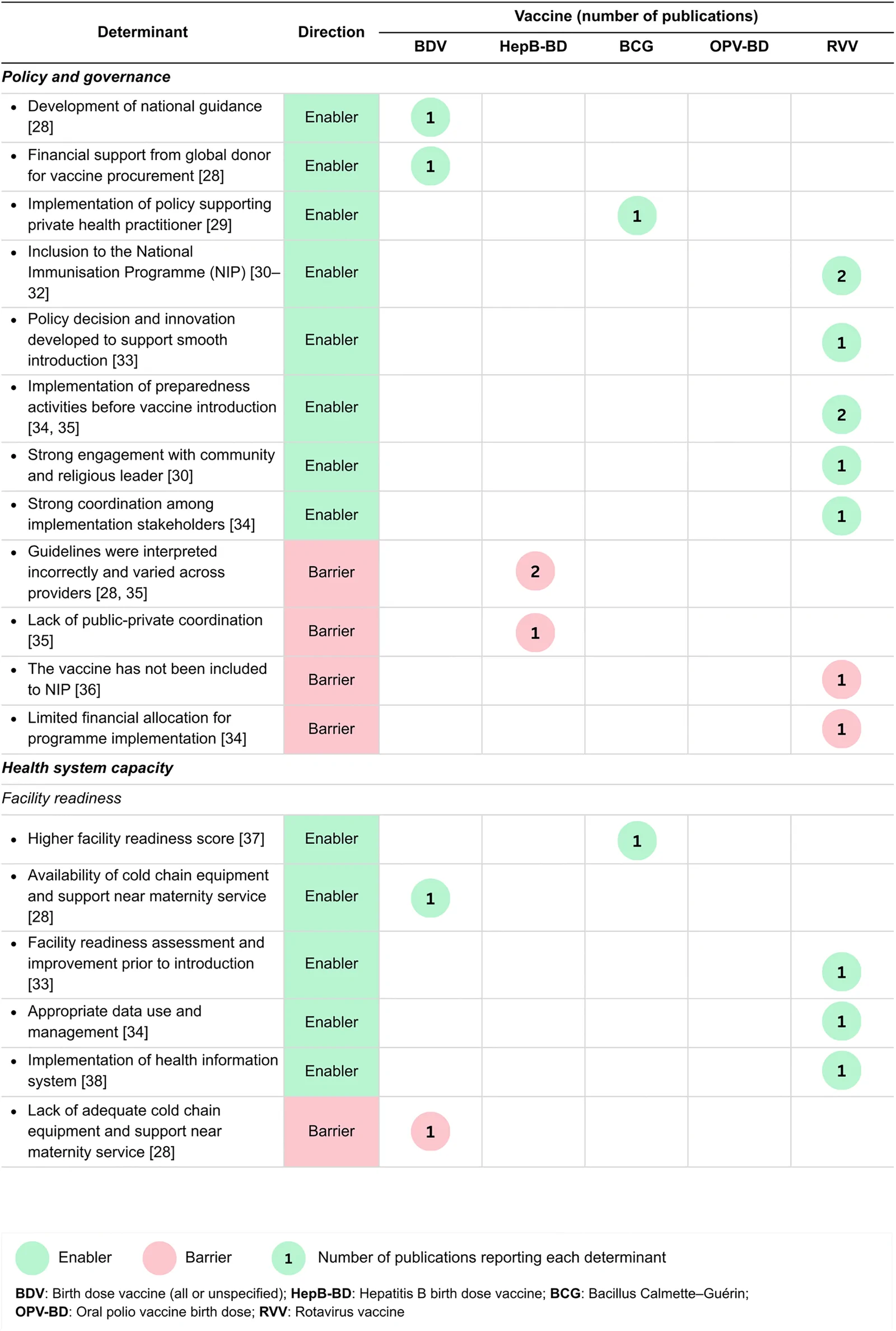
Supply-side enablers and barriers to BDV and RVV implementation. The number within each circle indicates the number of studies reporting the determinant for the respective vaccine. Green circles indicate enablers, whereas red circles indicate barriers. The number of studies reflects the breadth of reported evidence and does not indicate the magnitude or statistical significance of the determinant.

**Fig 4:**
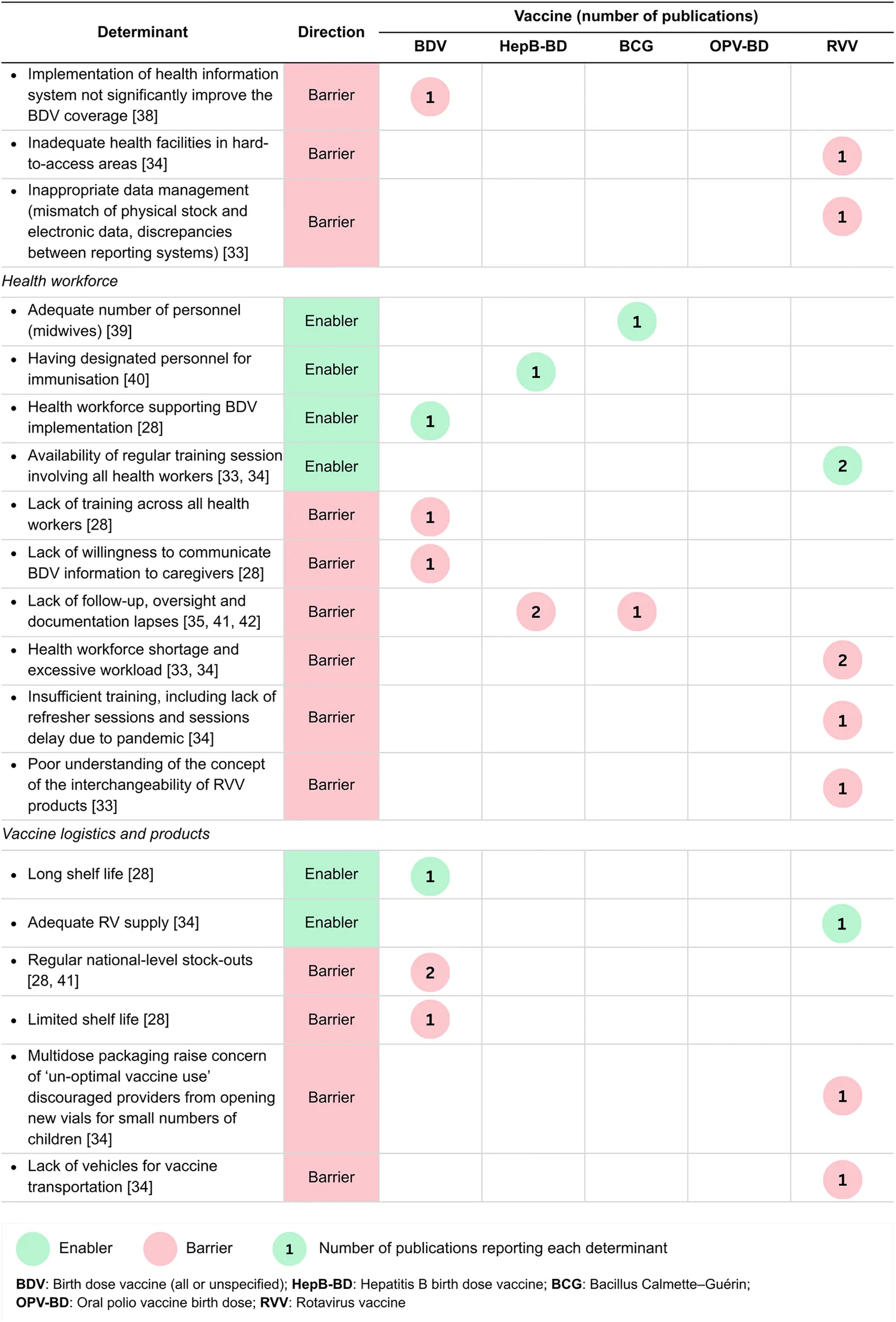
Supply-side enablers and barriers to BDV and RVV implementation (continued). The number within each circle indicates the number of studies reporting the determinant for the respective vaccine. Green circles indicate enablers, whereas red circles indicate barriers. The number of studies reflects the breadth of reported evidence and does not indicate the magnitude or statistical significance of the determinant.

**Fig 5:**
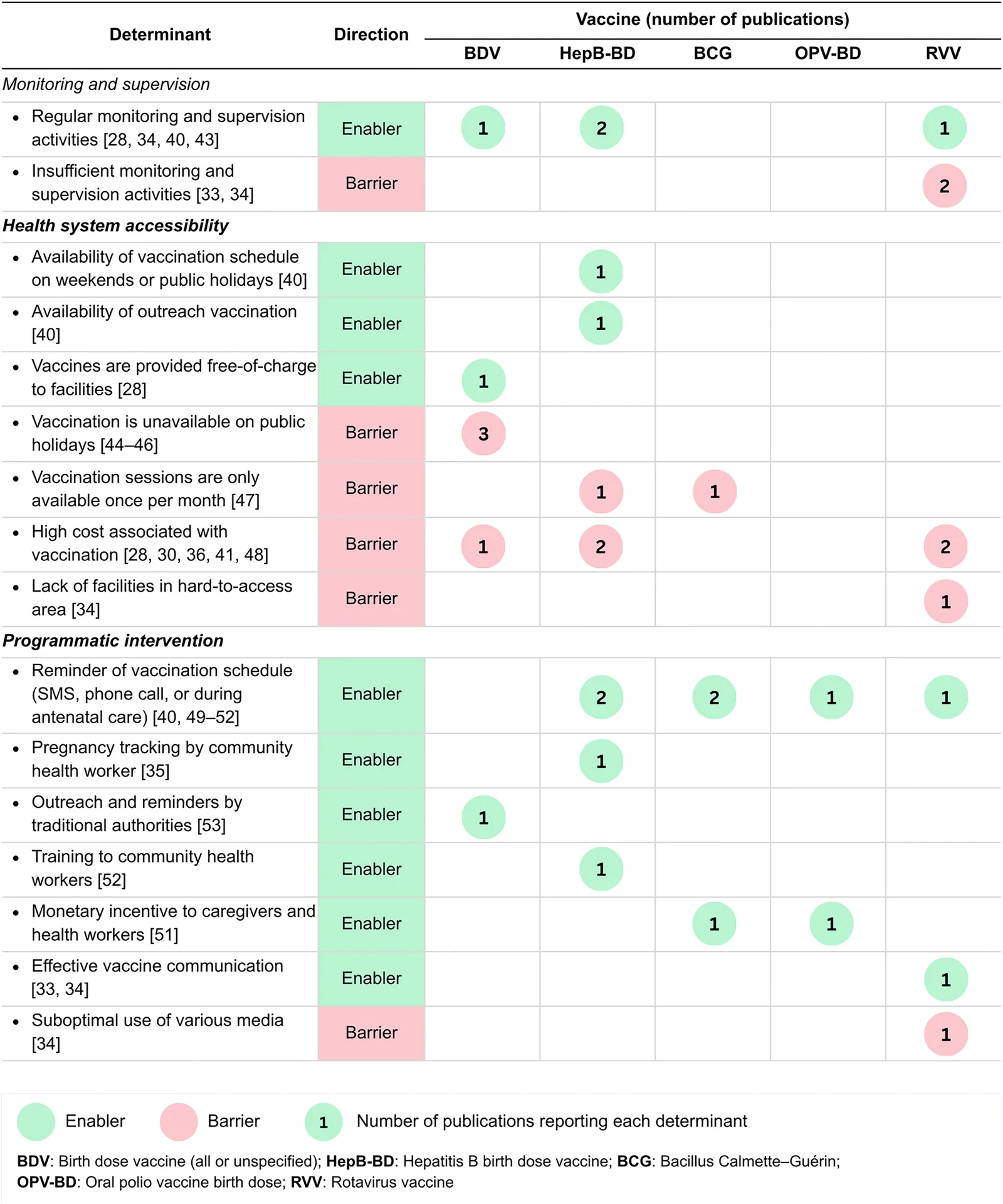
Supply-side enablers and barriers to BDV and RVV implementation (continued). The number within each circle indicates the number of studies reporting the determinant for the respective vaccine. Green circles indicate enablers, whereas red circles indicate barriers. The number of studies reflects the breadth of reported evidence and does not indicate the magnitude or statistical significance of the determinant.

1. Policy and governance

Across both BDV and RVV implementations, enablers and barriers were identified at the policy and governance level.

The existence of clear and coherent national guidelines is a key driver or enabler of timely BDV administration. In the Democratic Republic of Congo (DRC), national immunisation guidelines support decision-making at facility levels [28]. External support from international development partners such as Global Alliance for Vaccines and Immunization (GAVI) and UNICEF in strengthening logistics, such as overcoming storage and quality constraints by supplying vaccines and solar-powered refrigerators to facilities facing electricity outages and limited cold chain infrastructure, was viewed as an enabler of vaccine implementation in this country [28]. In Afghanistan, policies that facilitate coordination between public and private providers were associated with increased uptake by expanding the number of facilities offering BDV. The support also includes training, supply chain assistance and monthly incentives associated with higher coverage in their catchment areas [29].

Inclusion in the national immunisation programme (NIP) was a major driver of RVV implementation [30–32]. During the introduction phase, supportive policies that enabled smooth rollout were found to be effective, as demonstrated in a study from India [33]. These were complemented by preparedness activities, such as comprehensive planning, training, and operational innovations, to facilitate implementation [33,34]. Effective coordination among stakeholders involved in the vaccination programme was critical to these efforts [34]. Additionally, a study in Indonesia showed engagement with community and religious leaders helped increase public trust and vaccine acceptance by emphasising the severity of rotavirus disease and the government’s commitment to its prevention [30].

Despite these enablers, several technical barriers to BDV implementation were identified. Studies in the DRC and Vietnam reported that guidelines were often unclear and not uniformly implemented across facilities, resulting in inconsistent delivery of the BDVs [28,35]. In Vietnam, weak coordination between the public and private sectors was a key challenge, particularly when births occurred in private facilities while vaccination services were available only in the public sector. The large hospitals developed internal guidelines that deviated from national policy, while local health authorities lacked the power to enforce compliance [35].

Only two studies reported RV implementation barriers in this domain. RVV non-inclusion in NIP was identified as a major barrier to achieving high uptake, as suggested by an Indonesian study conducted prior to RV inclusion in NIP [36]. A study from India reported limited financial allocation resulted in limited financial and logistical support as well as a lack of human resources [34].

2. Health system capacity
  a. Facility readiness

A high level of facility readiness was identified as a key facilitator of BDV implementation. Findings in the DRC showed that the probability of BCG vaccination within 24 hours was found to be higher in facilities with higher WHO-assessed readiness scores [37]. In the same setting, the availability of small refrigerators as vaccine storage located near delivery wards and solar panels to ensure a consistent power supply was found as an enabler in ensuring timely uptake [28]. Similar factors were identified for RVV. Multilevel cold chain assessment and augmentation prior to RVV introduction in India were identified as vital for smooth implementation [33]. Beyond cold chain capacity, drivers include the implementation of health information systems for coverage reporting and stock management, alongside the appropriate use of data for programme management and decision-making, as demonstrated in Ethiopia and India [34,38].

In the operational readiness sub-domain, barriers to BDV implementation were reported in two studies. In the DRC, a country representing a low-resource setting, barriers persist in the form of inadequate facility resources, including BDV being stored in immunisation wards distant from the place of delivery, limited space for storage, and unreliable electricity supply [28]. Unintuitive results were found in Ethiopia, where health information systems (HIS) are often considered an enabler in vaccination programmes; their implementation was found to be an insignificant improvement of BDV uptake, potentially reflecting missed vaccinations in private practices that are not routinely recorded [38].

Similarly, insufficient facility readiness continues to hinder effective RVV implementation, particularly in hard-to-reach areas with limited resources [34]. Data management challenges were also reported, including mismatches between electronic and physical stock records, as well as discrepancies arising from fragmented reporting systems [33].

b. Health workforce

As the primary implementers of vaccination programmes, the health workforce capacity was identified as a key facilitator of BDV delivery. A study in Ghana showed that a higher number of midwives per clinic was associated with increased BCG uptake [39]. In Papua New Guinea, facilities with designated staff for HepB-BD vaccination were more likely to achieve higher timely coverage than those without [40]. In a qualitative study in the DRC, support from health workers was driven by evidence of BDV effectiveness and recommendations from the Expanded Programme on Immunisation (EPI) as well as global guidance (e.g., WHO guidelines), strengthening their confidence in delivering vaccination programmes [28].

For RVV, the availability of capacity-building activities targeting health workers was identified as crucial for effective implementation. In India, a qualitative study highlighted training sessions supported by development partners as particularly useful [34]. Key aspects included training on cold chain management and electronic systems, as well as the involvement of all stakeholders engaged in programme implementation prior to vaccine introduction [33,34].

However, barriers to BDV implementation persist, including insufficient training and health worker attitudes. In the DRC, training that targeted only designated vaccination providers, without including all relevant staff, particularly delivery midwives, was identified as a barrier to achieving timely BDV [28]. Additional gaps at the health worker level were also reported in multiple settings, including inadequate communication of the benefits and importance of vaccination to mothers in the DRC [28], ineffective pregnancy tracking to identify eligible infants in Vietnam [35], poor documentation of maternal HBsAg status in delivery ward records in Ghana [41], and limited follow-up after immunisation in India [42]; all were associated with reduced timeliness or uptake.

In RVV implementation, the lack of refresher sessions and delayed training were reported as key barriers in India [34]. Shortages of health workers, both in overall numbers and in appropriate task allocation, were also identified as critical challenges in the same setting [33,34]. Additionally, gaps in knowledge were noted, including limited understanding of issues such as the interchangeability of vaccine products [33].

c. Vaccine logistics and product

Multidose formulations of Hep-B BD with a long shelf life (a 28-day post-opening) were perceived as an enabler for BDV implementation in DRC [28]. In India, RVV implementation was facilitated by consistent vaccine availability [34].

Barriers to BDV implementation included recurrent vaccine stockouts at both national and facility levels, as reported in DRC and Ghana [28,41]. A study in the DRC also reported that multidose vial packaging introduced additional logistical complexity. BCG vials with 20 doses viable for less than six hours after opening and the need for 10–20 simultaneous newborns to justify opening a single vial created practical difficulties in low-volume facilities [28].

In RVV implementation, similar challenges were observed. In India, multidose packaging created reluctance among providers to open new vials unless full use could be ensured [34]. Additional barriers included limited transport capacity for vaccine distribution, which was associated with poor delivery performance [34].

d. Monitoring and supervision

Regular supervision and monitoring activities were reported as facilitators for uptake and timeliness of both BDV and RVV uptake in both low-income and middle-income settings [28,34,40,43]. Identified barriers included limited supervision in hard-to-reach areas, high workloads among immunisation officers, and insufficient supervisory support across several settings [33,34].

3. Health system accessibility

Schedule availability and vaccination costs were classified as supply-side factors influencing access. In Papua New Guinea, facilities with available staff for vaccination on weekends were associated with a higher proportion of timely BDV [40]. Another enabler identified was the provision of vaccines free of charge to facilities in the DRC [28]. The provision of outreach vaccination linked to facilities and the availability of HepB-BD at health facilities were associated with higher timely uptake in Papua New Guinea [40].

Barriers of BDV implementation, identified from Nigeria and the Philippines, included limited schedules, and often inaccessible on weekends or public holidays [44–46]. In the Philippines, primary health care facilities offering immunisation sessions only once per month were associated with delayed vaccination compared with facilities offering weekly sessions [47]. Cost-related barriers were reported in Ghana and Uganda, where the high cost associated with the vaccines posed a challenge for equal access [41,48], as well as in the DRC, where additional costs associated with vaccination (e.g., for vaccination cards) were reported as too high to afford [28]. In addition, the non-transferability of cards across facilities and complicated immunisation tracking also become a barrier in the DRC [28].

For RVV, no facilitators related to access were identified from the included studies. However, in Indonesia, prior to NIP inclusion, the high price of the rotavirus vaccine was perceived as beyond the financial reach of most parents, effectively limiting uptake to higher-income households [30,36]. This finding reinforces the transformative role of NIP inclusion, not only as a governance decision but also as a direct mechanism for removing financial barriers and ensuring equitable access. The other critical gap, the hard-to-reach geographic areas, lacked adequate health facilities to deliver RV reported in India. This finding, from Assam State in India, highlights the disproportionate burden borne by populations in remote areas who face both physical access barriers and insufficient facility infrastructure, a dual challenge that is particularly acute for vaccines requiring multiple doses across the first year of life [34].

4. Programmatic intervention

Programmatic interventions, including reminders, beneficiary tracking and outreach, health worker capacity-building, and incentives, were reported as facilitators of BDV uptake. Reminder-based approaches with educational messages were among the most consistently evidenced: regular SMS reminders in Nigeria, phone call reminders to caregiver in Ghana, and mHealth interventions delivered during antenatal and postnatal care in Ethiopia [49–51], all successfully improved uptake in low-resource settings. Informing mothers about HepB-BD during antenatal care visits was associated with improved coverage in Papua New Guinea [40], and the development of an antenatal birth-dose plan by caregivers was associated with higher HepB-BD timeliness in Kiribati [52]. Tracking and outreach conducted by both community health workers and traditional authorities (including barbers as community outreach agents) were also linked to increased uptake and timeliness of BDV in Vietnam and Nigeria [35,53]. Training community health workers to counsel caregivers on vaccination was effective in Kiribati [52]. Monetary incentives for both caregivers and community health volunteers also demonstrated an impact on timely BCG and OPV1 coverage in Ghana, where small financial rewards upon verified on-time vaccination yielded measurable improvements [51].

During its early introduction, RVV implementation was supported by the effectiveness of targeted vaccine communication delivered in immunisation session sites and effective outreach interventions. In a study in India, these included the consistent delivery of four key messages to every caregiver at session sites, monthly Village Health and Nutrition Day sessions, and targeted sensitisation of mothers and pregnant women addressing fear of vaccines; ease of access at government facilities; protection against diarrhoea; and the vaccine’s palatable taste were identified as enabling factors for RV uptake [34]. Additional strategies reported in this setting comprised the display of IEC materials in multiple languages, pre-introduction communication on vaccine rollout, and coordinated communication through media sensitisation, mass campaigns, and interpersonal engagement by frontline health workers [33,34]. Outreach interventions, such as house visits combined with message delivery to raise RVV awareness, were also reported [34]. A key barrier identified in this study was the suboptimal use of media to remind caregivers of the vaccination schedule [34].

### Demand-side determinants

Demand-side enablers and barriers of BDV and RVV implementation are summarised in Figs 6–10.

**Fig 6:**
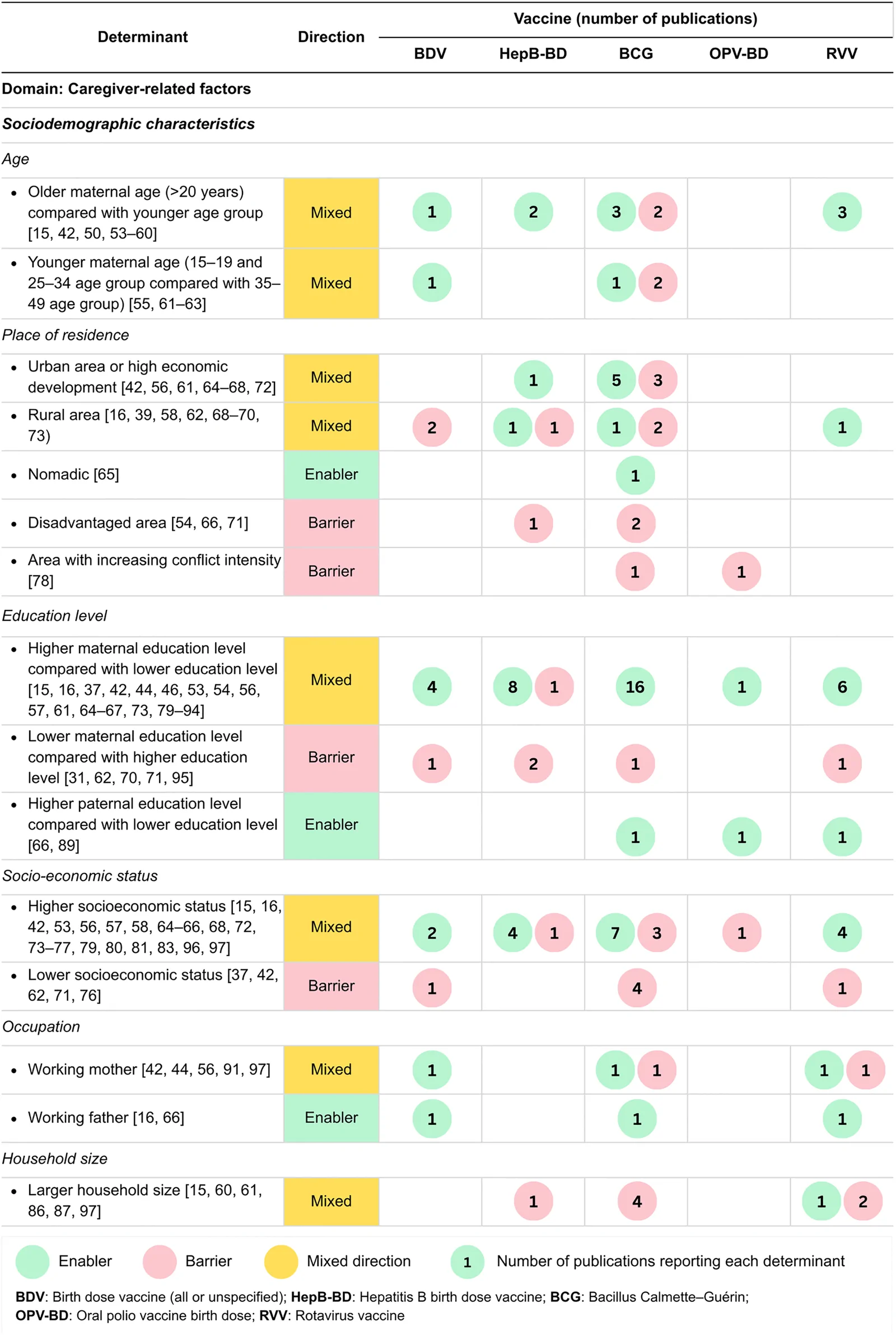
Demand-side enablers and barriers to BDV and RVV implementation. The number within each circle indicates the number of studies reporting the determinant for the respective vaccine. Green circles indicate enablers, whereas red circles indicate barriers. The number of studies reflects the breadth of reported evidence and does not indicate the magnitude or statistical significance of the determinant.

**Fig 7:**
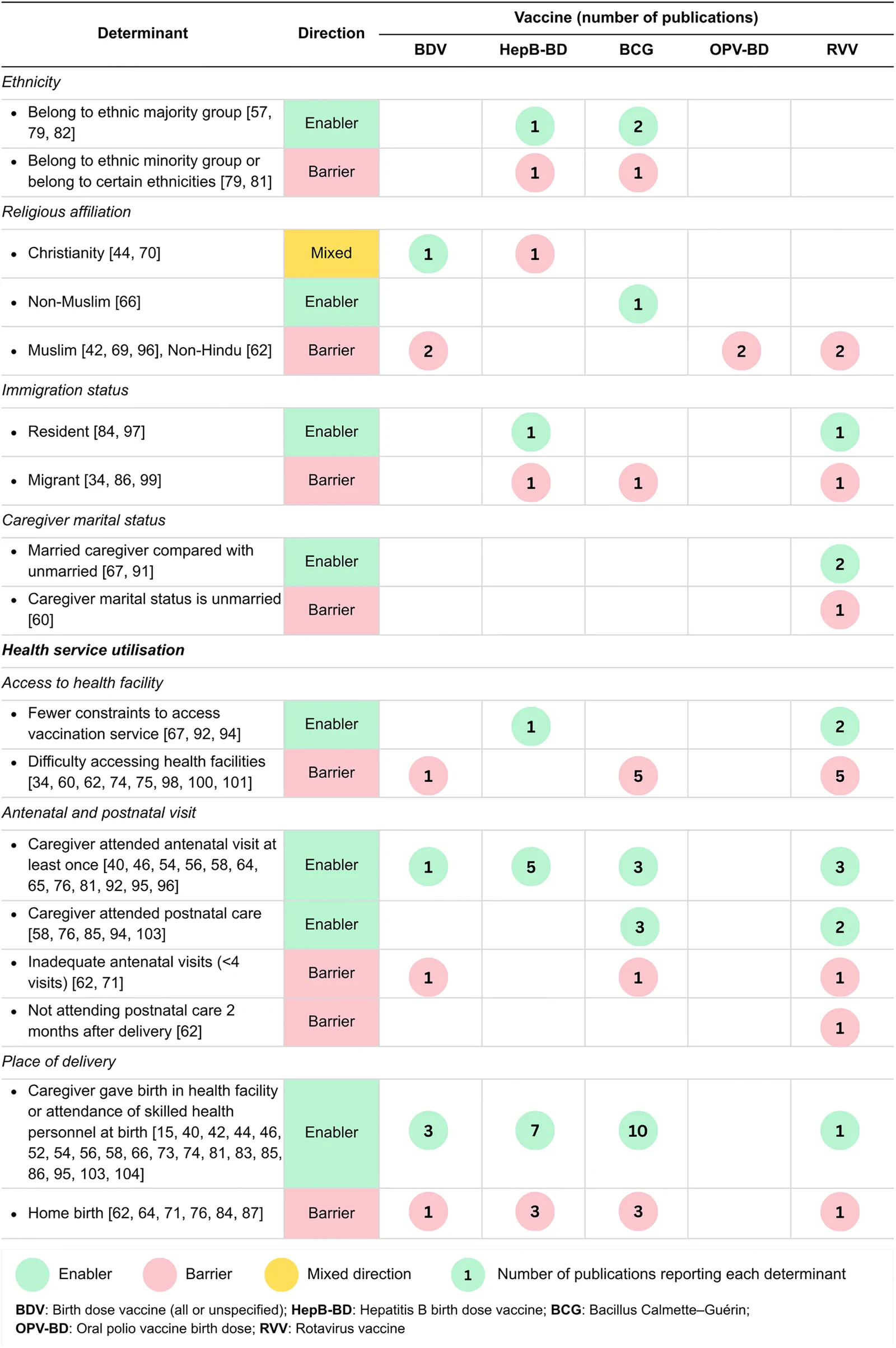
Demand-side enablers and barriers to BDV and RVV implementation (continued). The number within each circle indicates the number of studies reporting the determinant for the respective vaccine. Green circles indicate enablers, whereas red circles indicate barriers. The number of studies reflects the breadth of reported evidence and does not indicate the magnitude or statistical significance of the determinant.

**Fig 8:**
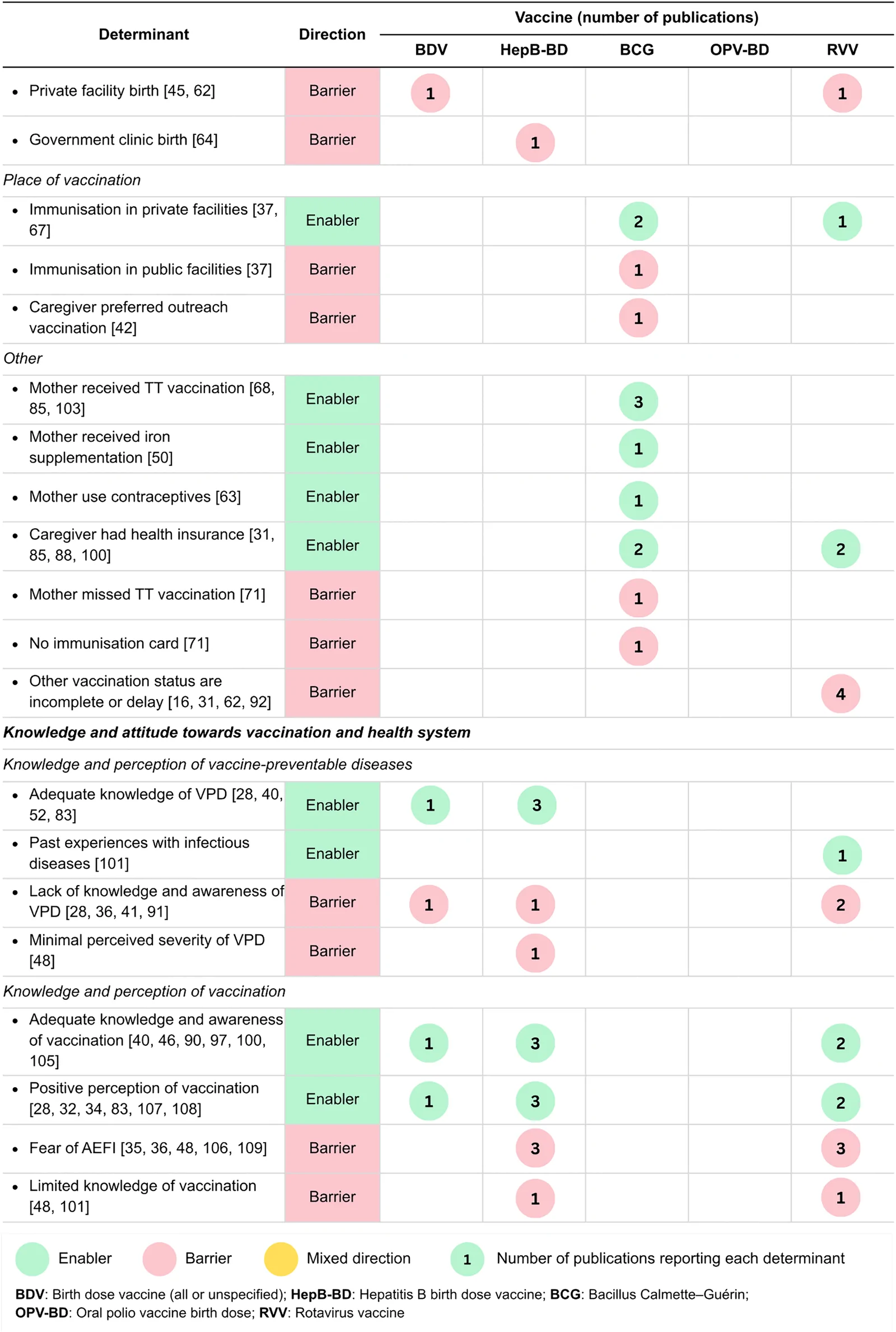
Demand-side enablers and barriers to BDV and RVV implementation (continued). The number within each circle indicates the number of studies reporting the determinant for the respective vaccine. Green circles indicate enablers, whereas red circles indicate barriers. The number of studies reflects the breadth of reported evidence and does not indicate the magnitude or statistical significance of the determinant.

**Fig 9:**
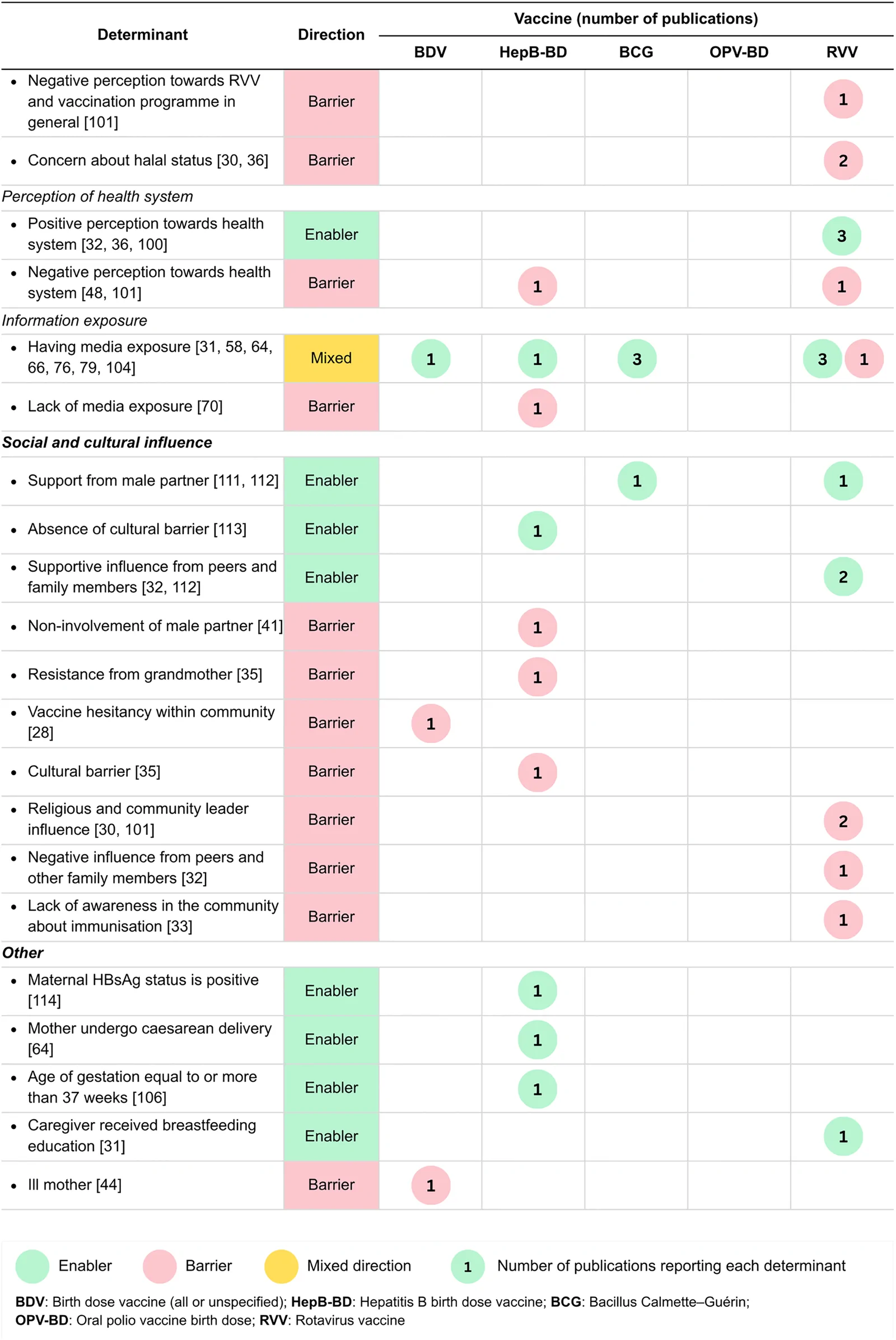
Demand-side enablers and barriers to BDV and RVV implementation (continued). The number within each circle indicates the number of studies reporting the determinant for the respective vaccine. Green circles indicate enablers, whereas red circles indicate barriers. The number of studies reflects the breadth of reported evidence and does not indicate the magnitude or statistical significance of the determinant.

**Fig 10:**
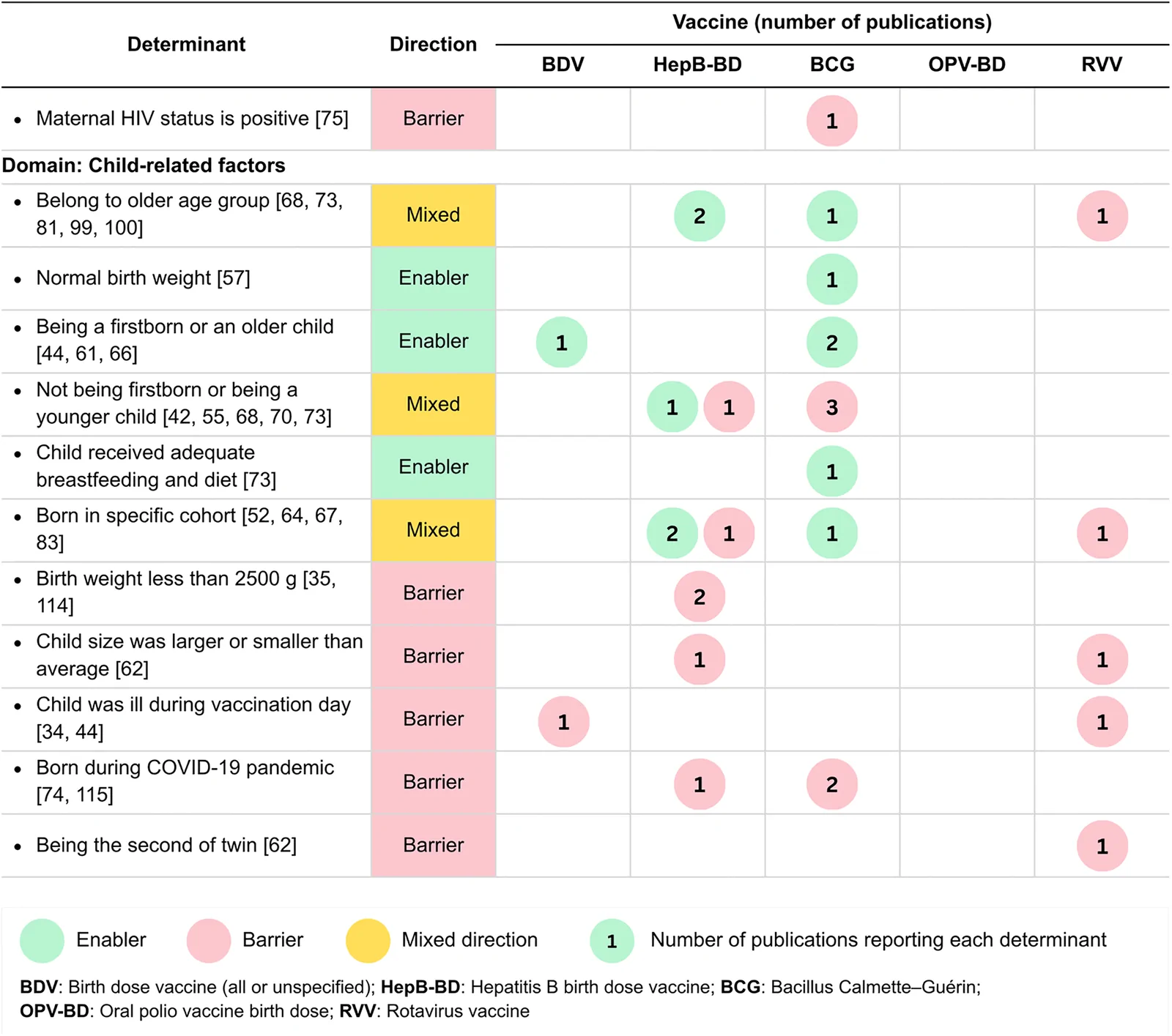
Demand-side enablers and barriers to BDV and RVV implementation (continued). The number within each circle indicates the number of studies reporting the determinant for the respective vaccine. Green circles indicate enablers, whereas red circles indicate barriers. The number of studies reflects the breadth of reported evidence and does not indicate the magnitude or statistical significance of the determinant.

1. Caregiver-related factors
  a. Socio-demographic characteristics
    i. *Caregiver’s age*

In both BDV and RVV, maternal age was associated with vaccination uptake and timeliness. Across several cohorts in Asia and Africa, children of older mother groups, typically aged over 20, 30, or 35 years, were more likely to receive timely BDVs or RVV [50,53–60]. Conversely, younger maternal age, particularly among adolescents (≤19 years), was consistently associated with missed doses or untimely vaccination [55,61].

However, two studies, one originating from India and a multinational study encompassing Bangladesh, Brazil, India, Nepal, and Peru, reported contrasting trends, with lower timeliness observed among older maternal age groups [15,42]. Other variations were also noted, including higher BDV uptake among mothers aged 15–19 in India (ref. 45–49) [62] and 25–34 years in Indonesia (ref. 35–49) [63], indicating that the relationship may differ across settings.

ii. *Place of residence*

Analysis of national and multinational data found that residence in urban areas or regions with higher levels of economic development was associated with higher BDV uptake and timeliness [56,61,64–67]. In contrast, children living in rural or disadvantaged areas were more likely to experience lower uptake or delayed vaccination for both BDVs [16,54,62,66,68–71] and RVV [58]. However, contrasting findings were also observed, with studies from India, Somalia, and Gambia reporting lower uptake in urban areas [42,68,72] and higher uptake and timeliness in rural settings observed in India and Ghana [39,73]. In Somalia, nomadic populations were reported to have higher uptake compared with urban and rural populations [65].

Variations across administrative regions were also reported in Senegal, India, Guinea-Bissau, Myanmar, and El Salvador, although the underlying reasons were not specified [55,62,64,67,73–77]. Additionally, one study in Nigeria identified residence in areas with higher conflict intensity as a barrier to BDV vaccination [78].

iii. *Education level*

Twenty-seven studies identified higher maternal education as a determinant of increased BDV and RVV uptake and timeliness [15,16,37,42,44,46,53,54,56,57,61,64–67,79–94], while lower maternal education was associated with a greater likelihood of delay or missed doses, as reported in five studies [31,62,70,71,95]. Paternal education was also found to be positively associated with uptake for both BDV and RVV [66,89] Only one study reported an inverse trend, where higher education was associated with lower uptake [73]; however, no studies found that lower education was linked to higher uptake.

iv. *Socioeconomic status*

An association between higher socioeconomic status, commonly measured by a higher wealth index, and improved uptake and timeliness was reported in sixteen studies on BDV [15,16,53,56,57,64–66,68,72,74–76,80,81,83] and four studies on RVV [16,58,96,97]. Lower socioeconomic status, in contrast, defined by a lower wealth index [37,62,71,76] or belonging to the lowest caste [42], was associated with reduced uptake and delayed vaccination in five studies. However, four studies reported an inverse association, with a higher wealth index linked to lower uptake, suggesting context-specific variation in this relationship [42,73,77,79].

v. *Occupation*

Few studies identified parental occupation as a determinant of BDV and RVV uptake. Formal paternal employment was consistently associated with higher uptake for both vaccines [16,66]. In contrast, the association between maternal employment and uptake was mixed: studies from Ethiopia and Nigeria reported a positive association of uptake and working mothers [44,56,91], whereas studies from India and China found a negative association [42,97].

vi. *Marital status, relationship with child, and household size*

Marital status was found significantly associated with RVV, with conflicting results [60,67,91], but no significant association was found in BDV studies. Being the father of the child was associated with higher uptake, but only assessed in one study [93]. Larger household size was generally associated with lower uptake of both BDV [15,61,86,87] and RVV [60,97]. Only one study reported higher uptake among children from larger households [60].

vii. *Ethnicity*

Children from ethnic majority households were generally more likely to receive timely vaccination [57,79,82], while those from ethnic minority groups were more likely to miss doses [79,81]. Some studies also reported lower uptake among specific ethnic groups with lower levels of health system engagement, although others did not identify clear explanations for these disparities [72,98].

viii. *Religious affiliation*

Religious affiliation of caregivers was identified as a factor associated with BDV and RVV uptake in some settings. In India, non-Hindu groups, such as Muslims and Christians, were reported to have lower uptake [42,62,70]. In contrast, in Nigeria, children from Christian households had higher uptake compared with those from Muslim households [44]. A negative association between Muslim affiliation and vaccination uptake was also reported in Burkina Faso, Ethiopia, and Bangladesh [66,69,96].

ix. *Immigration status*

Children with non-migrant caregivers were more likely to receive BDV [84] and RVV [97], whereas lower odds of vaccination were observed among children of immigrant caregivers [34,86,99].

b. Health service utilization
  i. *Access to health facilities*

Access to health facilities, such as close proximity [92], shorter travel time [94], and availability of transportation [67], was associated with higher uptake and timeliness of both BDVs and RVV. Identified barriers included long distances to facilities [60,74,75,98,100], time constraints [34,100,101], and difficulties in accessing healthcare (e.g., reporting major problems in reaching services) [62]. A study in Southern Tanzania also reported vaccination schedules coinciding with the rainy season further hindered access [98].

Additional determinants within this domain included preference for government health services, which was associated with BDV delays [42]; uncertainty about access to healthcare, identified as a barrier [102]; and recent contact with the health system, such as visiting a health facility within the past six months, which was associated with higher BDV uptake [63].

ii. *Antenatal and postnatal visit*

The number of antenatal visits was associated with the uptake and timeliness of both BDVs and RVV. Compared with children whose caregivers did not attend any antenatal visits, those whose caregivers attended at least one visit were more likely to receive BDV [40,46,56,64,65,76,81,95] or RVV [58,92,96]. One study further reported that attending at least four antenatal visits was associated with a higher likelihood of timely BDV uptake [54]. In other studies, attending fewer than four antenatal visits was associated with lower uptake of both BDV [62,71] and RVV [62]. Several aspects of antenatal care were also associated with higher uptake and timeliness. In a study from Papua New Guinea, increased uptake was linked to recommendations for facility-based delivery and the provision of information on HepB-BD during antenatal visits [40].

Postnatal care attendance was also associated with higher uptake and timeliness. Studies from India, Senegal, and Ethiopia showed that children whose caregivers attended postnatal care were more likely to receive timely vaccination than those who did not [58,76,85,94,103]. For BDV in particular, early contact with the health system after birth, such as postnatal check-ups following home delivery [56] and newborn weighing [54], was linked to higher uptake. Consistently, lack of contact with the health system after birth was identified as a barrier to the implementation of both BDV and RVV [62]. However, one study from Senegal reported a negative association between postnatal hospitalisation and uptake [104].

iii. *Place of delivery*

Institutional delivery was consistently associated with higher uptake and timely receipt of BDV [15,40,42,44,46,52,54,56,66,73,74,81,83,85,86,95,103,104] and RVV [58] across many settings, while home delivery was linked to missed doses or delayed administration [62,64,71,76,84,87]. Additionally, an analysis of data from 20 LMICs found that attendance by a skilled health professional at birth was associated with increased BDV uptake [61].

However, comparisons between public and private facilities showed mixed findings. Some studies reported higher uptake in public or province-level hospitals [105] and lower uptake in private facilities [45,62], whereas others found lower uptake in government clinics [64], indicating context-specific variation.

iv. *Place of vaccination*

In several contexts, the place of vaccination was associated with BDV and RVV receipt. Vaccination in private facilities was linked to higher uptake for BDVs [37,67] and RVV [67], whereas immunisation delivered through public facilities or outreach services was associated with lower uptake in some settings [37,42].

v. *Other*

Several other aspects of health service utilisation were also associated with BDV uptake. Mothers who received the tetanus toxoid (TT) vaccination [68,71,85,103], iron supplementation during pregnancy [50], and used contraceptives [63] were more likely to have their children vaccinated on time. Health insurance coverage was similarly linked to higher uptake [85,88], while lacking an immunisation card was associated with lower uptake [71]. Caesarean delivery [64] and gestational age of 37 weeks or more were also identified as determinants [106], although there are limited studies reporting this association.

A similar pattern was observed for RVV, where broader health system engagement predicted uptake. Children who had incomplete or delayed uptake of other routine vaccines were less likely to receive RVV [16,31,62,92], whereas having health insurance coverage was associated with higher uptake of RVV [31,100].

c. Knowledge and perception of vaccination
  i. *Knowledge and perception of vaccine-preventable diseases*

Many studies identified limited knowledge and low perceived severity of vaccine-preventable diseases (VPDs) as barriers to the implementation of both BDVs [28,41,48] and RVV [36,91]. Conversely, good knowledge and higher perceived severity were identified as implementation drivers [40,52,83,101].

ii. *Knowledge and perception of vaccination*

Positive awareness and perceptions of vaccination services were identified as key enablers of BDV [28,40,46,83,90,105,107,108] and RVV implementation [32,34,97,100], while limited knowledge and awareness posed important challenges [48,101]. Favourable perceptions included viewing vaccines as a means to protect children from infection, recognising their effectiveness in reducing disease burden, and attributing aspirational value to vaccination [32,34]. For BDV specifically, maternal confidence in vaccinating newborns at birth was an important driver of uptake [108].

Negative perceptions included fear of adverse events following immunisation (AEFI) [35,36,48,106,109], concerns about halal status [30,36], and suspicion toward vaccines, particularly in the context of COVID-19, where vaccination was perceived by some as having ulterior motives [101]. Mandatory vaccination policies were sometimes viewed as coercive, and the introduction of multiple vaccines within a short period was also perceived as a barrier [101].

One study using a discrete choice experiment (DCE) examined caregiver preferences for vaccine attributes. Overall, caregivers preferred vaccines with higher effectiveness, longer duration of protection, a low risk of mild side effects, and minimal out-of-pocket costs [110].

iii. *Perception of health system*

Negative perceptions of the health system were identified as barriers to BDV [48] and RVV programmes [101], particularly in the form of mistrust arising from ineffective communication between healthcare workers and caregivers. While no enablers were identified for BDV within this domain, positive perceptions, stemming from high overall service satisfaction [100], trust in healthcare provider recommendations [36], and strong relationships with health workers, were reported as facilitators of RVV implementation [32].

iv. *Information exposure*

Exposure to information, particularly through media, was generally associated with higher uptake of BDV [64,66,76,79,104] and RVV among children [58], while lack of media exposure was linked to non-receipt [70]. The source of information also influenced uptake. One study from Lebanon found that caregivers who primarily received information from nurseries or schools had lower uptake compared with those who obtained information mainly from health facilities [99]. An finding in Peru, however, showed lower uptake associated with having TV at home [31].

d. Social and cultural influence

In terms of social and cultural influences, male partner involvement was associated with uptake of both BDV and RVV. Support from male partners, such as attending antenatal care, was linked to a higher likelihood of children receiving BDV or RVV [111,112], while their absence was identified as a barrier [41]. Other family influences included grandmothers, who in some cases opposed BDV due to perceptions that healthy newborns did not require vaccination [35].

At the community level, negative peer influence [32,33] and vaccine hesitancy [28] were identified as barriers, whereas community support facilitated uptake [32,112]. Certain cultural practices, such as keeping newborns at home during the first month after delivery, posed barriers to timely vaccination [35], while the absence of such cultural barriers facilitated uptake [113]. Community and religious leaders were identified as potential enablers, particularly through their endorsement of vaccination programmes [30,101].

2. Child-related factor

Most BDV studies found that being a firstborn child was associated with higher uptake [44,61,66], while later birth order was linked to lower uptake [42,55,68,70]. However, one study reported a contrasting finding, where non-firstborn children were more likely to receive the birth dose [89]. Normal birth weight was associated with higher BDV uptake [57], whereas low birth weight [35,114] or birth size perceived as smaller or larger than average [62] was linked to lower uptake. No enablers or barriers related to birth order or birth weight were identified in RVV studies.

Child age showed mixed associations with vaccination outcomes. In some studies, older age was associated with higher uptake [68,81,99], while in others it was linked to delayed or missed vaccination, indicating a potentially negative effect on timeliness [73,100].

Several additional child-related and temporal factors were identified. Enablers included breastfeeding with an adequate diet [73]. Higher uptake was also reported among children born during programme implementation periods [52] or after vaccine introduction into national immunisation schedules [83], as well as in certain birth cohorts [67]. In children with HIV, HIV-exposed uninfected or unknown status, early infant diagnosis, and being alive at the time of assessment were associated with higher uptake [82].

Barriers included child illness on the day of vaccination [34,44], being the second of twins [62], and being born during the COVID-19 pandemic [74,115]. Some studies also reported lower uptake in specific birth cohorts and delivery years [64,67].

## Discussion

This review highlights multiple enablers and barriers to BDV and RVV implementation across both supply and demand domains, both of which have the potential to influence the future implementation of the birth-dose RVV. In addition, factors related to new vaccine introduction or the switching of established vaccine products and schedules may collectively intertwine. These findings are particularly relevant to LMICs considering birth-dose RVV introduction or transition, where programme implementation may depend on the capacity of existing immunisation and birth-dose delivery platforms.

### Supply-side enablers and barriers

Identified factors in the supply side, mostly from qualitative studies, reveal that BDV and RVV implementation is shaped by multi-level factors spanning supportive policy, adequate health system capacity, and effective service delivery. Previous evidence highlighted the association of good governance and vaccination uptake [116] and that vaccination uptake requires both a comprehensive and adaptive approach across all implementation levels [117]. For the upcoming birth-dose RVV switching, programmatic issues related to birth dose platform and existing RVV in particular are both pivotal.

While inclusion in NIP was identified as a potential enabler for equitable birth dose RVV access in LMICs, the identified determinants from existing RVV programmes imply that successful implementation requires extending to effective execution of programme introduction activities. In high-income settings, where high coverage was achieved across all countries with NIPs, inclusion alone did not correlate with higher coverage rates [118]. International donor support, such as GAVI, may help address resource and capacity constraints, particularly in settings with limited health-system capacity. However, such support is not uniformly available across LMICs, underscoring the need to strengthen sustainable domestic capacity while leveraging external resources where available [119,120]. As a lesson learned from BDV delivery, strengthening the supply side policy and infrastructure across both public and private health facilities, alongside providing early education, were identified as crucial to ensure timely vaccine delivery at birth. Strategies to ensure timely birth-dose delivery should also be tailored to the local context by leveraging existing enablers and strengths while addressing identified barriers [117]. Furthermore, engagement of community and religious leaders as a trustworthy information source in the community was also identified as a potential demand-generation strategy [121], and was found to be potentially cost-effective [122,123].

In the context of future birth dose RVV, switching to a new immunisation schedule or vaccine product may present additional implementation challenges beyond the reported factors. Previous experiences with polio vaccine switching highlight the importance of comprehensive preparation and a well-coordinated implementation process prior to transition, including careful planning and assessment, strengthening health workforce capacity, and efficient logistics management [124]. In LMICs, where existing health-system constraints can affect the consistency of routine immunisation delivery, the maturity of planning and execution during the transition is essential to ensure that the switching process does not introduce additional barriers that could undermine the original objectives of the switch.

### Demand-side determinants

The wide range of sociodemographic determinants identified in both BDVs and RVV implementation hints at the presence of inequity in vaccination access, which may potentially affect the implementation of birth dose RVV in a similar way. While several determinants showed consistent directions as enablers or barriers across studies, others demonstrated divergent associations. These differences may reflect context-specific variation in how sociodemographic factors shape vaccination access and implementation, highlighting areas that warrant further assessment when considering the feasibility and implementation of birth-dose RVV in different settings.

Among the determinants, maternal education level, socioeconomic status, and older maternal age were the most reported. Evidence also highlights associations between those characteristics and either uptake or timeliness of other childhood vaccinations and is also present in HICs [125]. Socioeconomic status serves as a predictor for both vaccination access and health literacy, including vaccination-related knowledge [126]. Maternal education is found to be associated with lower hesitancy and higher health literacy [127], although they are not always associated with knowledge of specific vaccine-preventable diseases in particular [128]. A notable, unsurprising finding was elucidated from multiple qualitative studies, reporting that caregivers often did not understand the specific details of the antigen or benefits of vaccination, a phenomenon that also occurred in a high-income setting [129]. This finding suggests that general vaccination knowledge may be sufficient to encourage participation, particularly when multiple vaccines are administered during the same visit, but may be insufficient to sustain uptake amid circulating misinformation and hoaxes. In addition, general knowledge may be less helpful in supporting catch-up vaccination when caregivers have limited understanding of specific vaccine antigens and the importance of missed doses, which may be addressed through reminder and recall tools that support tracking incomplete or missed vaccinations while potentially reducing gaps across educational and socioeconomic groups [130].

Nevertheless, reducing inequities also requires context-adapted supply-side interventions to ensure service accessibility for vulnerable populations. Strategies including service integration with other maternal and child health interventions to reduce visits [131] or expanded vaccination service availability in both place and schedule to create multiple points of access [29,132,133] could bridge existing access gaps. In the BDV context, birth in health facility, antenatal and postnatal care utilisation were found to be consistently associated with higher uptake, implying a potential strategy to improve vaccination coverage in the perinatal period, as also implied by previous research [134,135].

Despite the overall trends, several determinants were reported to have conflicting directions, as illustrated by lower maternal education, lower socioeconomic status, and younger maternal age. A previous study suggested that a sociodemographic characteristic may be associated with different vaccination outcomes across settings due to its complex interactions with other determinants and health behaviours [136]. For example, although high income is linked with adequate access in most settings, there is a possibility that in some settings, caregivers from higher-income households are more likely to vaccinate their children in private facilities, where recording and reporting systems may be less well established [79]. Therefore, these contrasting findings warrant the need for further research to better understand the context-specific variations.

The findings from the demand-side indicate that factors such as knowledge, misinformation, trust, family decision-making, and sociocultural considerations may also influence the uptake of birth-dose RVV in LMICs. However, the manifestation and relative importance of these factors may vary across settings, reflecting differences in sociocultural norms, health-seeking practices, information environments, and trust in health services. This suggests that demand-generation strategies for birth-dose RVV should build on existing community engagement and communication approaches while being adapted to local contexts. Rather than assuming that determinants identified in one setting will apply uniformly across LMICs, understanding context-specific concerns and information needs will be important for developing approaches that support informed and timely acceptance of birth-dose vaccination.

### Strengths and limitations

This review specifically focused on the BDV and RVV context rather than childhood immunisation in general. Although several identified determinants may also apply to other childhood vaccines, the review provides specific insights into BDV delivery that may inform efforts to strengthen BDV systems. In addition, the review captured broad supply- and demand-side drivers and determinants across a range of study designs, allowing comprehensive identification of enablers and barriers to BDV and RVV implementation. The inclusion of evidence from diverse settings, particularly low- and middle-income countries, also broadened the contextual relevance of the findings.

The study has several limitations. For quantitative studies, only statistically significant determinants were reported, based on the rationale that only factors significantly associated with coverage or timeliness could be identified as enablers or barriers. As a result, non-significant findings were not included in the synthesis [137–139]. Although a substantial number of quantitative studies were identified, cumulative effect estimates could not be generated because no meta-analysis was conducted. Although the number of studies reporting each determinant varied, with some determinants being reported more frequently than others, these frequencies should not be interpreted as reflecting their relative importance, as not all determinants were assessed across all studies. In addition, the review may be subject to publication and language bias, as only studies retrieved from the selected databases and published in English or Indonesian were included. Results were synthesised from available evidence predominantly from India, Indonesia, China, Ethiopia, and Nigeria, with limited representation of literature from other regions, such as Central and South America. Therefore, the findings may over-represent these countries and may not directly reflect programmatic or sociocultural determinants in under-represented countries.

We also identify gaps in research assessing supply-side determinants, highlighting potential future assessment in this field. Despite the direct influence of supply-side factors on vaccination uptake, currently most of them were explored from qualitative studies, while there is still a lack of study quantifying their association. Further research on supply-side factors is needed, as these were less frequently examined in the included studies. Future studies could explore supply-side determinants across different contexts, given potential variation in implementation drivers, as well as evaluate interventions aimed at improving health system capacity and service accessibility. Quantitative studies are also needed to assess the magnitude of identified supply-side enablers and barriers. In addition, future research should move beyond identifying determinants alone to examining how these factors influence immunisation behaviour and vaccine uptake more broadly. In the context of introducing an RVV birth dose, original research assessing programme readiness and context-specific enablers and barriers is needed to inform effective implementation.

## Conclusion

Implementation of BDV and RVV is influenced by both supply- and demand-side determinants. On the supply side, identified enablers and barriers encompassed policy and governance, health system capacity, health service accessibility, and programmatic interventions. On the demand side, higher maternal education, higher socioeconomic status, health facility birth, and antenatal-postnatal care attendance were consistently identified as major enablers, while home delivery, lower socioeconomic status, and residence in rural areas were commonly reported barriers. Further research is needed to better understand supply-side determinants, the mechanisms through which identified factors influence immunisation behaviour and uptake, and the readiness for future RVV birth-dose implementation across different contexts.

## Supporting information

S1 Appendix Supplementary files. (DOCX)

S1 File PRISMA Checklist. (DOCX)
