# Supplementary material for: Bridging the birth dose gap: A scoping review of implementation challenges for birth dose and rotavirus vaccines in low- and middle-income countries
Source: PLOS Glob Public Health. 2026 Nov 6;6(11):e0006910. doi: 10.1371/journal.pgph.0006910 (PMC13742884; doi:10.1371/journal.pgph.0006910)
Supplement: Supplementary file 1 [file pgph.0006910.s001.docx]

**Bridging the Birth Dose Gap: A Scoping Review of Implementation Challenges for Birth Dose and Rotavirus Vaccines in Low- and Middle-Income Countries**

# Supplementary Files

**Table 1.** Keywords used for literature search

| **Search Date** | **Database** | **Keywords** | **Number of Results** |
| --- | --- | --- | --- |
| 13/02/2025 | Portal Garuda | (imunisasi) AND (orang tua OR ibu) AND (faktor) | 481 |
| 03/03/2026 | Portal Garuda | (imunisasi) AND (orang tua OR ibu) AND (faktor) | 76 |
| 05/03/2026 | PubMed | (vaccin*[tiab] OR immuni*[tiab] OR rotavirus[tiab] OR "hepatitis B"[tiab] OR polio[tiab] OR BCG[tiab] OR "birth dose"[tiab]) AND (child*[tiab] OR infant*[tiab] OR newborn*[tiab]) AND (parent*[tiab] OR mother*[tiab] OR caregiver*[tiab]) AND (enabler*[tiab] OR barrier*[tiab] OR determinant*[tiab] OR hesitan*[tiab] OR accept*[tiab] OR refus*[tiab] OR uptake[tiab] OR coverage[tiab]) AND ("low income countr*"[tiab] OR "middle income countr*"[tiab] OR LMIC[tiab] OR UMIC[tiab] OR "developing countr*"[tiab] OR Afghanistan[tiab] OR Albania[tiab] OR Algeria[tiab] OR Angola[tiab] OR Argentina[tiab] OR Armenia[tiab] OR Azerbaijan[tiab] OR Bangladesh[tiab] OR Belarus[tiab] OR Belize[tiab] OR Benin[tiab] OR Bhutan[tiab] OR Bolivia[tiab] OR "Bosnia and Herzegovina"[tiab] OR Botswana[tiab] OR Brazil[tiab] OR "Burkina Faso"[tiab] OR Burundi[tiab] OR "Cabo Verde"[tiab] OR Cambodia[tiab] OR Cameroon[tiab] OR "Central African Republic"[tiab] OR Chad[tiab] OR China[tiab] OR Colombia[tiab] OR Comoros[tiab] OR "Democratic Republic of the Congo"[tiab] OR "Republic of the Congo"[tiab] OR "Costa Rica"[tiab] OR "Côte d'Ivoire"[tiab] OR Cuba[tiab] OR Djibouti[tiab] OR Dominica[tiab] OR "Dominican Republic"[tiab] OR Ecuador[tiab] OR Egypt[tiab] OR "El Salvador"[tiab] OR "Equatorial Guinea"[tiab] OR Eritrea[tiab] OR Eswatini[tiab] OR Ethiopia[tiab] OR Fiji[tiab] OR Gabon[tiab] OR Gambia[tiab] OR Georgia[tiab] OR Ghana[tiab] OR Grenada[tiab] OR Guatemala[tiab] OR Guinea[tiab] OR "Guinea-Bissau"[tiab] OR Haiti[tiab] OR Honduras[tiab] OR India[tiab] OR Indonesia[tiab] OR Iran[tiab] OR Iraq[tiab] OR Jamaica[tiab] OR Jordan[tiab] OR Kazakhstan[tiab] OR Kenya[tiab] OR Kiribati[tiab] OR Kosovo[tiab] OR Kyrgyzstan[tiab] OR Laos[tiab] OR Lebanon[tiab] OR Lesotho[tiab] OR Liberia[tiab] OR Libya[tiab] OR Madagascar[tiab] OR Malawi[tiab] OR Malaysia[tiab] OR Maldives[tiab] OR Mali[tiab] OR "Marshall Islands"[tiab] OR Mauritania[tiab] OR Mauritius[tiab] OR Mexico[tiab] OR Micronesia[tiab] OR Moldova[tiab] OR Mongolia[tiab] OR Montenegro[tiab] OR Morocco[tiab] OR Mozambique[tiab] OR Myanmar[tiab] OR Namibia[tiab] OR Nepal[tiab] OR Nicaragua[tiab] OR Niger[tiab] OR Nigeria[tiab] OR "North Macedonia"[tiab] OR Pakistan[tiab] OR "Papua New Guinea"[tiab] OR Paraguay[tiab] OR Peru[tiab] OR Philippines[tiab] OR Rwanda[tiab] OR Samoa[tiab] OR "Sao Tome and Principe"[tiab] OR Senegal[tiab] OR Serbia[tiab] OR "Sierra Leone"[tiab] OR "Solomon Islands"[tiab] OR Somalia[tiab] OR "South Africa"[tiab] OR "South Sudan"[tiab] OR "Sri Lanka"[tiab] OR Sudan[tiab] OR Suriname[tiab] OR Syria[tiab] OR Tajikistan[tiab] OR Tanzania[tiab] OR Thailand[tiab] OR "Timor-Leste"[tiab] OR Togo[tiab] OR Tonga[tiab] OR Tunisia[tiab] OR Turkey[tiab] OR Turkmenistan[tiab] OR Tuvalu[tiab] OR Uganda[tiab] OR Ukraine[tiab] OR Uzbekistan[tiab] OR Vanuatu[tiab] OR Vietnam[tiab] OR "West Bank and Gaza"[tiab] OR Yemen[tiab] OR Zambia[tiab] OR Zimbabwe[tiab]) | 3104 |
| 05/03/2026 | Cochrane | (vaccin* OR immuni* OR rotavirus OR hepatitis NEXT B OR polio OR BCG OR birth NEXT dose):ti,ab,kw AND (child* OR infant* OR newborn*):ti,ab,kw AND (parent* OR mother* OR caregiver*):ti,ab,kw AND (enabler* OR barrier* OR determinant* OR hesitan* OR accept* OR refus* OR uptake OR coverage):ti,ab,kw AND (low NEXT income NEXT countr* OR middle NEXT income NEXT countr* OR LMIC OR UMIC OR developing NEXT countr* OR Afghanistan OR Albania OR Algeria OR Angola OR Argentina OR Armenia OR Azerbaijan OR Bangladesh OR Belarus OR Belize OR Benin OR Bhutan OR Bolivia OR "Bosnia and Herzegovina" OR Botswana OR Brazil OR "Burkina Faso" OR Burundi OR "Cabo Verde" OR Cambodia OR Cameroon OR "Central African Republic" OR Chad OR China OR Colombia OR Comoros OR "Democratic Republic of the Congo" OR "Republic of the Congo" OR "Costa Rica" OR "Côte d'Ivoire" OR Cuba OR Djibouti OR Dominica OR "Dominican Republic" OR Ecuador OR Egypt OR "El Salvador" OR "Equatorial Guinea" OR Eritrea OR Eswatini OR Ethiopia OR Fiji OR Gabon OR Gambia OR Georgia OR Ghana OR Grenada OR Guatemala OR Guinea OR "Guinea-Bissau" OR Haiti OR Honduras OR India OR Indonesia OR Iran OR Iraq OR Jamaica OR Jordan OR Kazakhstan OR Kenya OR Kiribati OR Kosovo OR Kyrgyzstan OR Laos OR Lebanon OR Lesotho OR Liberia OR Libya OR Madagascar OR Malawi OR Malaysia OR Maldives OR Mali OR "Marshall Islands" OR Mauritania OR Mauritius OR Mexico OR Micronesia OR Moldova OR Mongolia OR Montenegro OR Morocco OR Mozambique OR Myanmar OR Namibia OR Nepal OR Nicaragua OR Niger OR Nigeria OR "North Macedonia" OR Pakistan OR "Papua New Guinea" OR Paraguay OR Peru OR Philippines OR Rwanda OR Samoa OR "Sao Tome and Principe" OR Senegal OR Serbia OR "Sierra Leone" OR "Solomon Islands" OR Somalia OR "South Africa" OR "South Sudan" OR "Sri Lanka" OR Sudan OR Suriname OR Syria OR Tajikistan OR Tanzania OR Thailand OR "Timor-Leste" OR Togo OR Tonga OR Tunisia OR Turkey OR Turkmenistan OR Tuvalu OR Uganda OR Ukraine OR Uzbekistan OR Vanuatu OR Vietnam OR "West Bank and Gaza" OR Yemen OR Zambia OR Zimbabwe ):ti,ab,kw | 372 |
| 05/03/2026 | Scopus | TITLE-ABS-KEY ( vaccin* OR immuni* OR rotavirus OR "hepatitis B" OR polio OR BCG OR "birth dose" ) AND TITLE-ABS-KEY ( child* OR infant* OR newborn* ) AND TITLE-ABS-KEY ( parent* OR mother* OR caregiver* ) AND TITLE-ABS-KEY ( enabler* OR barrier* OR determinant* OR hesitan* OR accept* OR refus* OR uptake OR coverage ) AND TITLE-ABS-KEY ( "low income countr*" OR "middle income countr*" OR LMIC OR UMIC OR "developing countr*" OR Afghanistan OR Albania OR Algeria OR Angola OR Argentina OR Armenia OR Azerbaijan OR Bangladesh OR Belarus OR Belize OR Benin OR Bhutan OR Bolivia OR "Bosnia and Herzegovina" OR Botswana OR Brazil OR "Burkina Faso" OR Burundi OR "Cabo Verde" OR Cambodia OR Cameroon OR "Central African Republic" OR Chad OR China OR Colombia OR Comoros OR "Democratic Republic of the Congo" OR "Republic of the Congo" OR "Costa Rica" OR "C&#244;te d&apos;Ivoire" OR Cuba OR Djibouti OR Dominica OR "Dominican Republic" OR Ecuador OR Egypt OR "El Salvador" OR "Equatorial Guinea" OR Eritrea OR Eswatini OR Ethiopia OR Fiji OR Gabon OR Gambia OR Georgia OR Ghana OR Grenada OR Guatemala OR Guinea OR "Guinea-Bissau" OR Haiti OR Honduras OR India OR Indonesia OR Iran OR Iraq OR Jamaica OR Jordan OR Kazakhstan OR Kenya OR Kiribati OR Kosovo OR Kyrgyzstan OR Laos OR Lebanon OR Lesotho OR Liberia OR Libya OR Madagascar OR Malawi OR Malaysia OR Maldives OR Mali OR "Marshall Islands" OR Mauritania OR Mauritius OR Mexico OR Micronesia OR Moldova OR Mongolia OR Montenegro OR Morocco OR Mozambique OR Myanmar OR Namibia OR Nepal OR Nicaragua OR Niger OR Nigeria OR "North Macedonia" OR Pakistan OR "Papua New Guinea" OR Paraguay OR Peru OR Philippines OR Rwanda OR Samoa OR "Sao Tome and Principe" OR Senegal OR Serbia OR "Sierra Leone" OR "Solomon Islands" OR Somalia OR "South Africa" OR "South Sudan" OR "Sri Lanka" OR Sudan OR Suriname OR Syria OR Tajikistan OR Tanzania OR Thailand OR "Timor-Leste" OR Togo OR Tonga OR Tunisia OR Turkey OR Turkmenistan OR Tuvalu OR Uganda OR Ukraine OR Uzbekistan OR Vanuatu OR Vietnam OR "West Bank and Gaza" OR Yemen OR Zambia OR Zimbabwe ) | 4470 |

**Table 2.** Summary of study characteristics

| **Characteristics** | **Number of all articles**  **(N = 93)** | **%** | **Number of BDV articles**  **(N = 73)** | **%** | **Number of RV articles**  **(N = 24)** | **%** |
| --- | --- | --- | --- | --- | --- | --- |
| **Vaccination** | | |  |  |  |  |
| BDV | 73 | 78.5 | NA |  | NA |  |
| RVV | 24 | 25.8 | NA |  | NA |  |
| **Domain** |  |  |  |  |  |  |
| Supply-side | 29 | 31.2 | 23 | 31.5 | 6 | 25.0 |
| Demand-side | 88 | 94.6 | 68 | 93.2 | 24 | 100.0 |
| **Determinant** |  |  |  |  |  |  |
| Enabler | 78 | 83.9 | 59 | 80.8 | 23 | 95.8 |
| Barrier | 61 | 65.6 | 46 | 63.0 | 18 | 75.0 |
| **Study origin** |  |  |  |  |  |  |
| Asia | 43 | 46.2 | 32 | 43.8 | 13 | 54.2 |
| Africa | 41 | 44.1 | 33 | 45.2 | 8 | 33.3 |
| Other | 9 | 9.7 | 8 | 11.0 | 3 | 12.5 |
| Multicontinent | 3 | 3.2 | 3 | 4.1 | 1 | 4.2 |
| South and Central America | 3 | 3.2 | 2 | 2.7 | 2 | 8.4 |
| Oceania | 2 | 2.2 | 2 | 2.7 | 0 | 0 |
| Caribbean | 1 | 1.1 | 1 | 1.4 | 0 | 0 |
| **World Bank income category (2025)** | | | | | | |
| Low-income country | 19 | 20.4 | 14 | 19.2 | 5 | 20.8 |
| Lower-middle income country | 50 | 53.8 | 42 | 57.5 | 12 | 50.0 |
| Upper-middle income country | 24 | 25.8 | 17 | 23.3 | 7 | 29.2 |
| **Population level** |  |  |  |  |  |  |
| Facility | 9 | 9.7 | 8 | 11.0 | 3 | 12.5 |
| Subnational | 40 | 43.0 | 27 | 37.0 | 12 | 50.0 |
| National | 40 | 43.0 | 35 | 47.9 | 7 | 29.2 |
| Multinational | 4 | 4.3 | 3 | 4.1 | 2 | 8.3 |
| **Study design** | | |  |  |  |  |
| Quantitative | 81 | 87.1 | 67 | 91.8 | 18 | 75.0 |
| Cross-sectional study | 69 | 74.2 | 58 | 79.5 | 15 | 62.5 |
| Cohort study | 4 | 4.3 | 2 | 2.7 | 2 | 8.3 |
| Quasi-experimental | 4 | 4.3 | 4 | 5.5 | 0 | 0.0 |
| Clustered RCT | 2 | 2.2 | 2 | 2.7 | 0 | 0.0 |
| Case-control study | 1 | 1.1 | 1 | 1.4 | 0 | 0.0 |
| Other | 1 | 1.1 | 0 | 0.0 | 1 | 4.2 |
| Qualitative | 8 | 8.6 | 3 | 4.1 | 5 | 20.8 |
| Mixed-methods | 4 | 4.3 | 3 | 4.1 | 1 | 4.2 |

**Table 3.** List of country included in the study

| **Country** | **Number of studies** | **World Bank Income Group (2025)** | **Part of multi- national studies** | **Country** | **Number of studies** | **World Bank Income Group (2025)** | **Part of multi- national studies** |
| --- | --- | --- | --- | --- | --- | --- | --- |
| Afghanistan | 1 | L | No | Haiti | 1 | LM | Yes |
| Angola | 1 | LM | Yes | Honduras | 2 | LM | Yes |
| Armenia | 1 | UM | Yes | India | 13 | LM | Yes |
| Bangladesh | 4 | LM | Yes | Indonesia | 12 | UM | Yes |
| Benin | 1 | L | Yes | Jordan | 1 | LM | Yes |
| Bolivia | 1 | LM | Yes | Kenya | 6 | LM | Yes |
| Burkina Faso | 2 | L | Yes | Kiribati | 1 | LM | No |
| Burundi | 1 | L | Yes | Kyrgyz Republic | 1 | LM | Yes |
| Cambodia | 2 | LM | Yes | Lao PDR | 1 | LM | No |
| Chad | 1 | L | Yes | Lebanon | 1 | LM | No |
| China | 9 | UM | No | Lesotho | 2 | LM | Yes |
| Comoros | 1 | LM | Yes | Liberia | 1 | L | Yes |
| Colombia | 1 | UM | Yes | Madagascar | 2 | L | Yes |
| Congo | 1 | LM | Yes | Malawi | 5 | L | Yes |
| Congo, Dem. Rep. | 3 | L | Yes | Maldives | 1 | UM | Yes |
| Côte d'Ivoire | 1 | LM | Yes | Mali | 1 | L | Yes |
| Dominican Republic | 2 | UM | Yes | Mauritania | 1 | LM | Yes |
| Egypt, Arab Rep. | 2 | LM | Yes | Myanmar | 2 | LM | Yes |
| El Salvador | 1 | UM | No | Namibia | 1 | UM | Yes |
| Ethiopia | 8 | L | Yes | Nepal | 2 | LM | Yes |
| Gabon | 1 | UM | Yes | Niger | 1 | L | Yes |
| Gambia, The | 3 | L | Yes | Nigeria | 11 | LM | Yes |
| Ghana | 5 | LM | Yes | Pakistan | 3 | LM | Yes |
| Guatemala | 1 | UM | Yes | Papua New Guinea | 1 | LM | No |
| Guinea | 1 | LM | Yes | Peru | 4 | UM | Yes |
| Philippines | 3 | LM | Yes | Timor-Leste | 1 | LM | Yes |
| Rwanda | 2 | L | Yes | Togo | 1 | L | Yes |
| Senegal | 7 | LM | Yes | Türkiye | 1 | UM | Yes |
| Sierra Leone | 1 | L | Yes | Uganda | 3 | L | Yes |
| Somalia, Fed. Rep. | 2 | L | No | Viet Nam | 5 | LM | No |
| South Africa | 4 | UM | Yes | Yemen, Rep. | 1 | L | Yes |
| Tajikistan | 1 | LM | Yes | Zambia | 1 | LM | Yes |
| Tanzania | 4 | LM | Yes | Zimbabwe | 2 | LM | Yes |

**Table 4.** Study mapping based on domain

| **No** | **Domain** | **Enabler** | | | | **Barrier** | | | |
| --- | --- | --- | --- | --- | --- | --- | --- | --- | --- |
|  |  | **Studies assessing BDV (n)** | **Studies assessing RV (n)** | **All studies (n)** | **All studies (%)** | **Studies assessing BDV (n)** | **Studies assessing RV (n)** | **All studies (n)** | **All studies (%)** |
| 1 | Policy and governance | 3 | 3 | 7 | 7.5 | 3 | 2 | 4 | 4.3 |
| 2 | Health system capacity | 5 | 3 | 8 | 8.6 | 4 | 2 | 7 | 7.5 |
| 3 | Health system accessibility | 2 | 0 | 2 | 2.2 | 7 | 2 | 9 | 8.6 |
| 4 | Programmatic intervention | 7 | 2 | 9 | 9.7 | 0 | 1 | 1 | 1.1 |
| 5 | Caregiver - sociodemographic characteristics | 39 | 12 | 48 | 51.6 | 27 | 8 | 34 | 36.6 |
| 6 | Caregiver - health service utilisation | 30 | 7 | 36 | 38.7 | 14 | 8 | 21 | 22.6 |
| 7 | Caregiver - knowledge and perception of vaccination and health system | 14 | 8 | 22 | 23.7 | 7 | 9 | 16 | 17.2 |
| 8 | Caregiver - social and cultural influence | 3 | 4 | 7 | 7.5 | 3 | 2 | 5 | 5.4 |
| 9 | Caregiver - other | 1 | 3 | 4 | 4.3 | 2 | 0 | 2 | 2.2 |
| 10 | Child-related factor | 16 | 1 | 16 | 17.2 | 12 | 4 | 15 | 16.1 |

**Table 5.** Individual characteristics of included studies

| **No** | **Author, year** | **Country, Continent** | **World Bank Income group (2025)** | **Sampling level** | **Study period** | **Study design** | **Vaccine assessed** | **Domain (Supply or demand or both)** | **Identified enablers or barriers** |
| --- | --- | --- | --- | --- | --- | --- | --- | --- | --- |
| 1 | Ng’oda et al., 2026 [102] | Kenya, Africa | LM | Sub-  national | 2021-  2023 | Cross-  sectional | BCG | Demand-  side | Enablers:   - Religious affiliation of caregiver is christian, compared with no religious affiliation - Inability to access routine healthcare services during COVID-19 pandemic in the last six months: "do not know" |
| 2 | Cárdenas et al., 2026 [79] | Peru, South America | LM | National | 2021-  2024 | Cross-  sectional | BCG | Demand-  side | Enablers:   - First-spoken language of caregiver is Quechua, compared with Spanish - Caregiver completed higher education, compared with no education - Having mobile phone, compared with no mobile phone   Barriers:   - First-spoken language of caregiver is others, compared with Spanish - Belong to family with higher wealth index, compared with poor |
| 3 | Nguyen et al., 2026 [100] | Vietnam, Asia | LM | Sub-  national | 2025 | Cross-  sectional | RV | Demand-  side | Enablers:   - Caregiver has high knowledge score of rotavirus and RVV - Caregiver use health insurance regularly - Higher overall service satisfaction   Barriers:   - Older child age - Financial barrier to RVV: Time cost for travel - Financial barrier to RVV: Time cost post-vaccination - Longer distance to the nearest health service |
| 4 | Hassan et al., 2025 [65] | Somalia, Africa | L | National | 2020 | Cross-  sectional | BCG | Demand-  side | Enablers:   - Mother's education - primary (compared with no education) - Mother's education - secondary (compared with no education) - Place of residence - urban (compared with rural area) - Place of residence - nomadic (compared with rural area) - Numbers of antenatal visits: 2-3, or ≥ 4 (compared with no ANC visits) - Wealth index - higher (compared with lowest) |
| 5 | Hidaya-  tullah BW et al., 2025 [80] | Indonesia, Asia | UM | National | 2017 | Cross-  sectional | BCG | Demand-  side | Enablers:   - Mother's education - higher (in urban population) - Contraceptive use (in urban population) - Wealth index - richer (in rural population) |
| 6 | Vedel et al., 2025 [74] | Guinea-  Bissau, Africa | L | Sub-  national | 2017-  2020 | Cross- sectional | BCG | Demand- side | Enablers:   - Born at health facility - Socioeconomic score - higher - Born at health facility - Higher socioeconomic score - 5   Barriers:   - Year of birth: Born during COVID-19 pandemic (2020) - Residing in Quinara, Tombali, Gabu, or Bafata - Child's year of birth: born during COVID-19 pandemic - Distance from village to health facility |
| 7 | Ogundele et al., 2025 [91] | Nigeria, Africa | LM | Facility | Not stated | Cross- sectional | RV | Demand- side | Enablers:   - Married mothers - Mothers with secondary and higher education - Formally employed mothers   Barriers:   - No previous experience of child diarrhea |
| 8 | Handebo et al., 2025 [58] | Ethiopia, Africa | L | National | 2019 | Cross- sectional | RV | Demand- side | Enablers:   - Mother age - older - Wealth quintile: Higher index - ANC attendance - Born in health facility - PNC attendance - Exposed to media   Barriers:   - Living in rural area |
| 9 | Hassan et al., 2025 [68] | Somalia, Africa | L | National | 2020 | Cross- sectional | BCG | Demand-  side | Enablers:   - Wealth quintile: middle - Child's age: older than 11 month - Maternal TT vaccination status   Barriers:   - Birth order: not being first child - Place of residence: urban - Place of residence: rural |
| 10 | Gattam et al., 2025 [62] | India, Asia | LM | National | 2019-  2021 | Cross- sectional | Hep-B BD, BCG, OPV BD, RV | Demand-  side | **BDV**  Enablers:   - Time of survey: post COVID   Barriers:   - Child size at birth: very large, very small, or unknown - Caregiver education: lower - ANC visits: less than 4 - Place of delivery: home or private facility - Distance from health facility: big problem or not a big problem - Religion: non-Hindu - Zone: Northeast, central, west - Residency: rural - Baby checked within 2 months of delivery: No - Child size at birth: smaller than average - Wealth index: lower   **RVV**  Enablers:   - Time of survey: post COVID   Barriers:   - Twin: 2nd of twin - Baby checked within 2 months of delivery: No - Child size at birth: very small - Mother education: lower - ANC visits: less than 4 - Place of delivery: home or private facility - Wealth index: lower - Religion: non-Hindu - Zone: Other than south - Baby checked within 2 months of delivery: No - Mother education: lower - ANC visits: less than 4 - Household wealth index: lower - Zone: other than South - Religion: non-Hindu - Vaccine dose at 6 weeks not given on time: Yes |
| 11 | Huang et al., 2024 [114] | China, Asia | UM | National | 2019-  2021 | Cross- sectional | Hep-B BD | Demand -side | Enablers:   - Mother's HBsAg status: positive (in premature infants)   Barriers:   - Birth weight: 2000-2500, <2000 (in premature infants) |
| 12 | Derqui et al., 2024 [16] | 54 Low- and Middle-  Income Countries | L, LM, UM | Multi-  national | 2010-  2023 | Cross- sectional | Hep-B BD, BCG, RV | Demand  -side | **BDV**  Enablers:   - Higher wealth quintile (ref. poorest) - Mothers' level of formal education: higher level (ref. none) - Husband's occupation: employed (ref. no work)   Barriers:   - Living in rural area (ref. urban)   **RVV**  Enablers:   - Higher wealth quintile (ref. poorest) - Mothers' level of formal education: higher level (ref. none) - Husband's occupation: employed (ref. no work)   Barriers:   - Vaccination delay in 1st dose |
| 13 | Bassoum et al., 2024 [54] | Senegal, Africa | LM | National | 2019 | Cross- sectional | Hep-B BD | Demand- side | Enablers:   - Age of mother ≥ 35 years - Mother education level: primary level - Number of ANC ≥ 4 - Place of delivery: health facility   Barriers:   - Area of residence: Center and North |
| 14 | Mehra et al., 2024 [34] | India, Asia | LM | Sub- national | 2021 | Qualita-  tive | RV | Supply- and demand- side | Enablers:   - Timely preparation of due list of beneficiaries by the planning unit - Community mobilizer at village level actively visit beneficiaries before village health nutrition day - Regular Village Health, Sanitation and Nutrition Committee (VHSNC) Meetings - Consistent delivery of four key messages to every caregiver at the session site. - Exhaustive training sessions and involvement of development partners - House visits by cadres to educate the community - Monthly VHND sessions, sensitization of mothers and pregnant women - Display for targeted IEC materials in multiple languages - Pre-introduction communication about vaccine rollout - Safety of the vaccine and positive impact of vaccination - Regular supervisory visits and monitoring - Appropriate use of quality data in programme management and decision making - Monthly review meetings and regular analysis of data for programmatic improvement - Good coordination among stakeholders (planning unit, cold chain handler, lady health visitor) - Adequate RV supply - Regular training to cold chain handler on using electronic logistic system - Improved efficiency in supply chain through electronic management system   Barriers:   - Hard-to reach areas are difficult to access - Hard-to reach areas do not have adequate health facilities - Workforce was not rationally distributed in hard-to reach areas, lead to excess burden - Need for refresher sessions to new health officers - Delayed training to community health workers due to pandemic - Sub optimal use of various media - Prevailing fear of opening new vials among service providers and ensuring its full usage - Migration - Unavailability of parents to bring child to session site - Child feeling unwell on vaccination day - Access issues to hard-to-reach areas limits monitoring and supervisory activities - High workload of district immunisation officers and planning unit due to involvement in other programmes - Limited financial allocation - Lack of vehicles for vaccine transportation - Shortage of human resources |
| 15 | Dekker et al., 2024 [81] | Lao PDR, Asia | LM | National | 2011-  2017 | Cross- sectional | Hep-B BD | Demand- side | Enablers:   - Wealth index quintile: second to richest (2011/2012 data) Mother’s education level: primary, secondary, higher (2011/2012 data) - Place of delivery (last live birth of mother in the last two years): public hospital, private hospital, private clinic, other (2011/2012 data) - ANC visits (last live birth of mother in the last two years): Yes, <3 and more than 3 (2011/2012 data) - Age (years) of child: 1 and 2 years (2011/2012 data) - Wealth index quintile: second to richest (2017 data) - Place of delivery (last live birth of mother in the last two years): PHC, public hospital, private hospital, private clinic, other (2017 data) - Age (years) of child: 1 years (2017 data)   Barriers:   - Ethno-linguistic group of household: Mon-Khmer and Hmong-Mien (2011/2012 data) - Ethno-linguistic group of household: Hmong-Mien (2017 data) |
| 16 | Olakunde et al., 2024 [95] | Nigeria, Africa | LM | Sub- national | 2016-  2018 | Cross- sectional | Hep-B BD | Demand- side | Enablers:   - Antenatal care: Yes - Place of delivery: Institutional   Barriers:   - Educational attainment: none or primary |
| 17 | Huda et al., 2024 [92] | Pakistan, Asia | LM | Sub- national | 2019 | Cross- sectional | RV | Demand- side | Enablers:   - Distance from EPI center - Mothers' education - Antenatal care services - Cleanliness of drinking water and hygiene counselling   Barriers:   - ORS for diarrhea - Immunisation status |
| 18 | Boisson-Walsh et al., 2023 [37] | DRC, Africa | L | Sub- national | 2016-  2020 | Cross- sectional | BCG | Supply- and demand- side | Enablers:   - Mother's education: higher - Facility readiness: higher - Facility visit: private and other   Barriers:   - Household wealth index: second - Facility visit: public |
| 19 | Das and Khan, 2023 [73] | India, Asia | LM | National | 2015-  2021 | Cross- sectional | Hep-B BD | Demand- side | Enablers:   - Birth order: not being first child - Place of delivery: Institutional - Place of residence: rural   Barriers:   - Child age: more than 12 months - Mothers' education: primary, secondary, higher - Wealth quintile: higher - Region: not in north region |
| 20 | Adjei et al., 2023 [41] | Ghana, Africa | LM | Sub- national | 2020 | Qualita- tive | Hep-B BD | Supply- and demand- side | Barriers:   - Mother’s Denial of Hepatitis B Seropositivity - Mother’s Ignorance of Hepatitis B’s Effect on the Newborn - Male Partners’ Non-Involvement in Post-Test Counselling - Financial accessibility - Vaccine availability - Midwives' oversight and documentation lapses |
| 21 | Ma et al., 2023 [110] | China, Asia | UM | Sub- national | 2022 | Discrete choice experi- ment | RV | Demand- side | Enablers:   - Vaccine effectiveness: higher - Protection duration: longer - Risk of mild side effects: rare - Out-of-pocket costs - Time required for vaccination: 2 hours (shorter) |
| 22 | Wang et al., 2025 [78] | Nigeria, Africa | LM | National | 2008- 2023 | Cross- sectional | BCG, OPV BD | Demand  side | Barriers:   - Living in area with increased conflict intensity |
| 23 | Das, 2024 [73] | India, Asia | LM | National | 2019-  2021 | Cross- sectional | BCG | Demand-  side | Enablers:   - Breastfeeding children who received an adequate diet - Mothers protected against neonatal tetanus - Institutional births - Postnatal care provided by healthcare professionals |
| 24 | Summan et al., 2023 [115] | India, Asia | LM | National | 2019- 2021 | Cross- sectional | BCG, Hep-B BD | Demand- side | Barriers:   - COVID-affected (Born in 2020) |
| 25 | Anderson et al., 2022 [64] | Myanmar, Asia | LM | National | 2011- 2016 | Cross- sectional | Hep-B BD | Demand- side | Enablers:   - Mother's higher education - Household wealth quintiles: middle, richer, richest - Urban area - Weekly media exposure: yes - Recommended ANC services: yes - Cesarean delivery   Barriers:   - Delivery year (2015 and 2016) - Geographic zone: Central and Coastal - Delivery location: government clinic or home delivery |
| 26 | Bassoum et al., 2022 [54] | Senegal, Africa | LM | Sub- national | 2020 | Cross- sectional | BCG, Hep-B BD, OPV BD | Demand- side | Enablers:   - Access to TV - Place of birth: health facility - Newborn weighing immediately at birth   Barriers:   - Hospitalization of newborn immediately at birth |
| 27 | Sitaresmi et al., 2022 [36] | Indonesia, Asia | UM | Sub- national | 2013 | Qualita- tive | RV | Supply- and demand- side | Enablers:   - Trust in HCP recommendation   Barriers:   - Respondents were not aware of the burden of diarrheal disease. - Respondents did not perceive their child’s susceptibility to acquiring diarrhea - Rotavirus diarrhea is an almost unknown disease - Concerns regarding rotavirus vaccine included not being on the Indonesian NIP - The "newness" of the vaccine - the potential for adverse events, - the cost of vaccine - The use of trypsin porcine in the vaccine production process |
| 28 | Blose et al., 2022 [59] | South Africa, Africa | UM | Sub- national | 2012- 2016 | Cross- sectional | RV | Demand- side | Enablers:   - Adult caregiver |
| 29 | Dhalaria et al., 2022 [42] | India, Asia | LM | National | 2015- 2016 | Cross- sectional | BCG | Supply- and demand- side | Enablers:   - Education of mother: higher - Delivery: private/government hospital   Barriers:   - No follow up by health provider - Urban residence - Religion: Muslim - Caste: scheduled (lowest) caste and scheduled (marginalized) tribe - Socio-economic index: higher - Age of mother: older than 25 years - Occupation of mother: agriculture or live stock - Birth order: second - Preferred system for treatment: government - Preferred service place for immunisation: outreach |
| 30 | Boisson et al., 2022 [28] | DRC, Africa | L | Sub- national | 2021 | Qualita- tive | BCG, OPV BD, Hep-B BD (planned introduc-tion) | Supply- and demand- side | Enablers:   - Evidence based effectiveness of BD vaccines - Advocacy by global players eg. WHO - BD vaccine free-of-charge to facilities - HepB-BD multi-dose vials viable < 28 days after opening - Many facilities reported small fridges near maternity ward - Some reported regular cold-chain supervision - Some facilities have solar power fridges to maintain cold chain in case of electricity outage - Mothers and informants expressed optimism for BD innovation - Plans for timely and streamlined BDs in the DRC underway - Donors help cover the cost of BD vaccines - Informants support BD vaccines if PEV- and research-backed - Health system top-down - informants would accept changes made to the national immunisation schedule - Mothers sought information about HBV and other diseases - Mothers were comfortable vaccinating newborns within 24-hours of delivery - Mothers did not report experiencing vaccine hesitancy themselves   Barriers:   - 1-10 doses packaging options of HepB-BD - BCG's 20-dose vials viable < 6 hours after opening - Facilities require mothers to pay fee for vaccines (vaccine card) - Infants' vaccine cards rarely accepted across facilities - BD vaccines often kept in immunisation ward and not near the maternity ward - Some small fridges were not used for BD vaccines/lacked space - Electricity outages pose risk to the cold-chain temperature - Many facilities must retrieve BD vaccines daily at nearby reference facility - Need for BD vaccine training across disciplines - Clearer facility guidance on timely & streamlined BDs required - Regular national stockouts of BCG vaccine - Need for stronger willingness by informants to communicate BD vaccine information to mothers - Vaccine availability did not influence mother's choice of facility - Most mothers were not aware of risk, transmission, or of vaccine-preventable diseases - Mothers reported vaccine hesitancy within their communities |
| 31 | Ibraheem et al., 2022 [44] | Nigeria, Africa | LM | Sub- national | 2019- 2020 | Cross- sectional | BCG, Hep-B BD, OPV BD | Supply- and demand- side | Enablers:   - Hospital delivery - Order of birth: first; second to fourth - Religion: christianity - Moders education level: tertiary - Occupation of mother: petty trader or nurses/teacher   Barriers:   - Access and availability: Weekend/public holiday - Mother-related cause: Ill mother - Baby related: Ill baby |
| 32 | Ji et al., 2022 [93] | China, Asia | UM | Sub- national | 2017- 2020 | Prospec-tive cohort | RV | Demand- side | Enablers:   - Caregiver relation: father - Education: some college or more   Barriers:   - Vaccine hesitant |
| 33 | Hossain et al., 2021 [66] | Bangla- desh, Asia | LM | National | 1993- 2014 | Cross- sectional | BCG | Demand- side | Enablers:   - Place of residence: urban - Religion: other - Birth order: 1 - Higher education - Husband education: higher - Father's occupation: professionals - Wealth index: higher - Delivery place at health facility - Having media exposure   Barriers:   - Region: Sylhet |
| 34 | Shen et al., 2021 [82] | China, Asia | UM | National | 2011- 2017 | Retro- spective cohort | BCG | Demand- side | Enablers:   - HIV status: HIV-exposed uninfected or unknown - Early infant diagnosis - Survival status: live - Han ethnicity - Education: higher |
| 35 | Vink et al., 2021 [29] | Afghanis- tan, Asia | LM | Sub- national | 2018- 2019 | Cross- sectional | BCG | Supply- side | Enablers:   - PHP program |
| 36 | Mutyoba et al., 2021 [48] | Uganda, Africa | L | Sub- national | 2018 | Qualita- tive | Hep-B BD | Demand- side | Barriers:   - Lack of knowledge about HepB-BD benefit and schedule - Concern that vaccine may introduce infection to newborn - Low perceived severity of the disease - Cost of HBV - Ineffective healthcare worker-mother communication - Healthcare workers mistrust |
| 37 | Oladepo et al., 2020 [49] | Nigeria, Africa | LM | Sub- national | 2017- 2019 | Quasi- experi- mental | BCG | Supply- side | Enablers:   - SMS reminder intervention |
| 38 | Levine et al., 2021 [51] | Ghana, Africa | LM | Sub- national | 2018- 2019 | Cluster- randomi-sed control- led trial | BCG, OPV BD | Supply- side | Enablers:   - Phone reminder - Incentive |
| 39 | Raguindin et al., 2020 [47] | Philippi- nes, Asia | LM | National | 2011- 2017 | Cross- sectional | BCG, Hep-B BD | Supply- side | Barriers:   - Frequency of immunisation in the PHC: once a month (rare) |
| 40 | Dougherty et al., 2020 [53] | Nigeria, Africa | LM | Sub- national | 2017- 2018 | Quasi- experi- mental mixed- methods | BCG, Hep-B BD, OPV BD | Supply- and demand- side | Enablers:   - Received referral card from barber - Wealth status: middle (OPV0 only) - Mother's age group: 20-34; 35-49 - Mother's education: primary and secondary+ |
| 41 | Alhassan et al., 2020 [39] | Ghana, Africa | LM | Sub- national | 2015- 2017 | Ecologi- cal study | BCG | Supply- and demand- side | Enablers:   - Number of midwives per clinic - Rural area |
| 42 | Kusnanto et al., 2020 [63] | Indonesia, Asia | UM | National | 2017 | Cross- sectional | BCG | Demand- side | Enablers:   - Maternal age: 25-34 - Contraceptive use: using - Health facility visits: yes |
| 43 | David et al., 2020 [45] | Nigeria, Africa | LM | Sub- national | 2017- 2018 | Cross- sectional | BCG, Hep-B BD, OPV BD | Supply- and demand- side | Barriers:   - Weekend/public holiday delivery, vaccination day restrictions, stockouts, prematurity - Private hospital delivery |
| 44 | Seror et al., 2020 [76] | Senegal, Africa | LM | National | 2017 | Cross- sectional | BCG | Demand- side | Enablers:   - Postnatal check-up within 3 months (in both group) - Region: west (in children with no vaccination card) - Household wealth quintile: richest (in children with vaccination card) - Mother's exposure to media (in children with no vaccination card) - Mother attended antenatal care (in children with no vaccination card)   Barriers:   - Home delivery (in both group) - Region: south (in children with no vaccination card) - Household wealth quintile: poorest (in children with vaccination card) |
| 45 | Wondimu et al., 2020 [96] | Ethiopia, Africa | L | National | 2016 | Cross- sectional | RV | Demand- side | Enablers:   - Household wealth - Antenatal care use: yes   Barriers:   - Religion: Muslim |
| 46 | Peretti- Watel et al., 2019 [55] | Senegal, Africa | LM | National | 2017 | Cross- sectional | BCG | Demand- side | Enablers:   - Mother's age: >30 - Region: West and Center   Barriers:   - Mother's age: 14-19 - Birth order: 4+ order |
| 47 | Huynh et al., 2020 [106] | Vietnam, Asia | LM | Facility | 2017- 2018 | Cross- sectional | Hep-B BD | Demand- side | Enablers:   - Age of gestation (≥ 37 weeks)   Barriers:   - Side effect after vaccination (fever, shock, death in premature infants) |
| 48 | Odeny et al., 2019 [111] | Kenya, Africa | LM | National | 2013 | Cross- sectional | BCG | Demand- side | Enablers:   - Male ANC attendance |
| 49 | Padmawati et al., 2019 [30] | Indonesia, Asia | UM | Sub- national | 2018 | Qualita- tive | RV | Supply- and demand- side | Enablers:   - Including vaccine into the national program - Endorsement from religious and community leader - Strong coordination of health providers and community/religious leader   Barriers:   - High cost of vaccine - Association of vaccine with porcine |
| 50 | Hang Pham et al., 2019 [105] | Vietnam, Asia | LM | Sub- national | 2017 | Cross- sectional | Hep-B BD | Demand- side | Enablers:   - Delivery in province-level hospital   Barriers:   - Not receiving information about benefit of HepB during pregnancy |
| 51 | Tsehay et al., 2019 [56] | Ethiopia, Africa | L | National | 2016 | Cross- sectional | BCG | Demand- side | Enablers:   - Maternal age: 20 or older (20-34; 35-49) - Residence: urban - Mothers' education level: primary (grade 1-8) - Mother's occupation: agriculture - Mother's occupation: manual/household worker - Wealth index: higher - Place of delivery: public sector - Place of delivery: NGO or others - Health checked after discharge/delivery at home: yes - Number of antenatal visits: at least 1 |
| 52 | Wangen et al., 2019 [83] | China, Asia | UM | Sub- national | 1997- 2011 | Cross- sectional | Hep-B BD | Demand- side | Enablers:   - Institutional births - Birth year 2002 and above - Mother's education level - True transmission route index - Income group 2 and higher - Perceived protection 2 or higher |
| 53 | Nguyen Si Anh et al., 2019 [57] | Vietnam, Asia | LM | National | Not repor-ted | Cross- sectional | Hep-B BD | Demand- side | Enablers:   - Low birth weight: No - Ethnicity: Kinh/Hoa - Mother's age: older than 20 - Mother's education: higher - Mother's wealth status: higher |
| 54 | Ibraheem et al., 2019 [46] | Nigeria, Africa | LM | Facility | 2016 | Cross- sectional | Birth Dose | Supply- and demand- side | Enablers:   - Hospital delivery - Correctly states NPI schedule - ANC attendance: yes - Mothers' education level: post secondary   Barriers:   - Weekend/public holiday delivery; friday evening delivery/discharge |
| 55 | Hu et al., 2018 [63] | China, Asia | UM | Sub- national | 1999- 2017 | Cross- sectional | Hep-B BD | Supply- and demand- side | Enablers:   - Children's imigration status: resident - Maternal education level: higher - Economic development level of resident areas: middle and high   Barriers:   - Home delivery |
| 56 | Khan et al., 2018 [77] | India, Asia | LM | National | 2015- 2016 | Cross- sectional | BCG | Demand- side | Enablers:   - Postnatal care - Institutional births - Neonatal tetanus - Women schooling 10+ years - Health insurance |
| 57 | Hu et al,. 2018 [83] | China, Asia | UM | Sub- national | 2014- 2015 | Cross- sectional | BCG | Demand- side | Enablers:   - Place of delivery: hospital - Maternal education level: higher than or completed senior middle school   Barriers:   - Immigration status: Migrant - Number of siblings: ≥ 3 |
| 58 | Echaiz et al., 2018 [31] | Peru, South America | UM | National | 2012 | Cross- sectional | RV | Supply- and demand- side | Enablers:   - Mother receiving breast-feeding education - RV included in NIP - Incomplete measles immunisation - Children has SIS (national insurance)   Barriers:   - Illiterate: unintended pregnancy - Incomplete polio, influenza, and pneumococcal immunisation - TV at home |
| 59 | Hu et al., 2017 [87] | China, Asia | UM | Sub- national | 2014 | Cross- sectional | BCG, Hep-B BD | Demand- side | Enablers:   - Mothers' education level: higher than or completed senior middle school   Barriers:   - Number of children in the surveyed household: ≥ 3 - Place of delivery: home |
| 60 | Li et al., 2017 [52] | Kiribati, Oceania | LM | National | 2014- 2015 | Quasi- experi- mental | Hep-B BD | Supply- and demand- side | Enablers:   - Intervention: 1) educating pregnant women; enhancing communications between health workers and VHVs - Timing of birth: during the project - Place of birth: hospital - Caregiver developed a birth dose plan - Knowledge that HepB may be transmitted through blood transfusion |
| 61 | Ntenda et al., 2017 [71] | Malawi, Africa | L | National | 2004 and 2010 | Cross- sectional | BCG | Demand- side | Barriers:   - Place of delivery: home or other (2004) - Mother's education: no formal education (2010) - Antenatal visits: 1 or none (2004) - immunisation card: no card or had a card but its wherebaout unknown (2004) - No TTI during pregnancy (2004) - Household wealth: poorest or middle (2010) - Geographical region: Central (2004) |
| 62 | Hoest et al., 2017 [15] | 8 Low- and Middle- Income Countries | LM | Multi-  national | 2009- 2014 | Prospec-tive cohort | BCG | Demand- side | Enablers:   - Years of maternal education: longer - Water, assets, maternal education, income index: higher - Place of birth: hospital   Barriers:   - Number of children in household: more - Maternal age: older |
| 63 | Mbengue et al., 2017 [77] | Senegal, Africa | LM | National | 2013- 2014 | Cross- sectional | BCG, OPV BD | Demand- side | Barriers:   - Wealth index: higher - Region: Fatick |
| 64 | George et al., 2016 [32] | India, Asia | LM | National | 2014- 2015 | Qualita- tive | RV | Supply- and demand- side | Enablers:   - Vaccine included in the UIP list - Good relationship with pediatrician and their recommendation - Supportive influence from peers and other family members - "Being safe rather than sorry" principles - Aspirational value for vaccines   Barriers:   - Negative influence from peers and other family members |
| 65 | Mvula et al., 2016 [60] | Malawi, Africa | L | Sub- national | 2012- 2014 | Prospec-tive cohort | RV | Demand- side | Enablers:   - Mothers' marital status: unmarried (RV1 only) - Mothers' age ≥ 40 (older) - Household size (persons) ≥ 7   Barriers:   - Distance to road ≥ 1.5 - Number of children <5 years in household ≥ 3 - Distance to road more than 1 km |
| 66 | Wiesen et al., 2016 [40] | Papua New Guinea, Oceania | LM | National | 2014 | Program evalua-  tion | Hep-B BD | Supply- and demand- side | Enablers:   - Facility: received supervision for BD - Facility: Facility give hepB BD - Facility: Designated person for hepB BD administration - Facility: Staff available to vaccinate on weekends - Facility: Inform mothers of hepB BD during antenatal care - Facility: Bring hepB to vaccinate home births - outreach vaccination - Caregiver: born in health facility - Caregiver: knowledge - heard of HepB - Caregiver: knowledge - Knows mother-to-child transmission routes - Caregiver: knowledge - Heard of hepatitis B vaccine - Caregiver: knowledge - Knows timing of BD - Caregiver: antenetal care - Had visited antenatal care - Caregiver: antenetal care - Recommended to deliver in health facilities - Caregiver: antenetal care - Told about hepatitis B BD during antenatal care |
| 67 | Odutola et al., 2015 [137] | The Gambia, Africa | L | Sub- national | 2011 | Cross- sectional | BCG | Demand-side | No significant enablers and barriers were reported |
| 68 | Hajizadeh et al., 2015 [61] | 20 low- and middle- income countries | LM | Multi- national | 2001- 2008 | Cross- sectional | BCG | Demand- side | Enablers:   - Birth order: older child - Mothers education - Urban - Attendance of skilled health personnel   Barriers:   - Mothers' age at birth: 19 or below (younger) - Household size (more person in the household) - Log GDP per capita |
| 69 | Onsomu et al., 2015 [88] | Kenya, Africa | LM | National | 2008- 2009 | Cross- sectional | BCG | Demand- side | Enablers:   - Education: primary, secondary - Health insurance |
| 70 | Hu et al., 2015 [96] | China, Asia | UM | Sub- national | 2014 | Cross- sectional | RV | Demand- side | Enablers:   - Duration of living in surveyed areas: more than or equal to 6 months - Family income per month > 500 USD - Mother's awareness of importance of vaccination: yes   Barriers:   - Siblings: more than or equal to 2 - Mothers' occupation: working |
| 71 | Payne et al., 2014 [72] | The Gambia, Africa | L | Sub- national | 2010 | Cross- sectional | BCG | Demand- side | Enablers:   - Ethnic group: Fula and Wolof - Wealth index   Barriers:   - Area of residence: urban and peri-urban |
| 72 | Suárez- Castaneda et al., 2014 [67] | El Salvador, Central America | LM | National | 2011 | Cross- sectional | BCG, RV | Demand- side | **BDV:**  Enablers:   - Urban area - Year of birth: born in 2006 and 2008 - Interviewee: is also the caregiver - Caregiver education: secondary education - Vaccinated in the private sector - Travels to the health centre by taxi   **RVV:**  Enablers:   - Interviewee: parent of the child - Caregiver marital status: married - Vaccinated in the private sector   Barriers:   - Place of residence: oriental region - Year of birth - Male |
| 73 | Schoeps et al., 2013 [69] | Burkina Faso, Africa | L | Sub- national | 2008- 2009 | Cross- sectional | BCG | Demand- side | Barriers:   - Religion: Muslim - Area of residence: rural |
| 74 | Le Polain de Waroux et al., 2013 [98] | Tanzania, Africa | L | Sub- national | 2004 | Cross- sectional | BCG | Demand- side | Barriers:   - Distance more than or equal to 5 km - Ethnicity: Mwera or Other - Distance more than or equal to 5 km - Seasonality: Rainy |
| 75 | Ndirangu et al., 2009 [75] | South Africa, Africa | UM | Sub- national | 2005- 2006 | Cross- sectional | BCG | Demand- side | Enablers:   - Wealth index   Barriers:   - Maternal HIV status - Distance to mobile clinic |
| 76 | Ameen et al., 2026 [109] | Nigeria, Africa | LM | Sub- national | 2024 | Cross- sectional | RV | Demand- side | Barriers:   - Safety - Side effects - Newness - Dosage |
| 77 | Gilano et al., 2025 [50] | Ethiopia, Africa | L | Sub- national | 2024 | Cluster- randomi-sed control- led trial | BCG | Supply- and demand- side | Enablers:   - mHealth intervention (SMS reminder of immunisation schedule) - Iron supplementation for mother - Mother's age: older |
| 78 | Phiri et al., 2025 [101] | Ethiopia, Kenya, Malawi, Africa | L, LM | Multi- national | 2023- 2024 | Qualita- tive | RV | Demand- side | Enablers:   - Past experiences with infectious disease - Perceived risks of non-vaccination - Engagement of trusted local stakeholders and religious leaders in educating communities   Barriers:   - Vaccination fatigue: perceived high frequency of vaccine campaigns - Suspicions surrounding COVID-19 vaccines - Perception of coercion - Limited knowledge about certain childhood vaccines - Difficult access to vaccination appointment - Negative perception of vaccine provider |
| 79 | Worku et al., 2024 [38] | Ethiopia, Africa | L | National | 2020- 2022 | Quasi- experi-  mental | BCG, RV | Supply- side | **BDV**  Barrier:   - Implementation of health information system   **RVV**  Enabler:   - Implementation of health information system |
| 80 | Kumar et al., 2024 [33] | India, Asia | LM | National | 2022 | Mixed- methods | RV | Supply- and demand- side | Enablers:   - Political willingness and strong government support - Well-planned preparedness activities - Operational innovation to facilitate smooth introduction and implementation - Training for all health workforce involved, completed prior to vaccine introduction - Multilevel cold chain assessment and space augmentation - Vaccine communication through media sensitization, mass communication, and interpersonal communication by frontline health worker   Barriers:   - Need for better supervision - Non-availability of updated microplan - Vaccine hesitancy - Mismatch of electronic stock data with physical stock - The concept of interchangeability is not well-understood - Multiple data sources result in discrepancies between the state and HMIS data reporting - Shortage of manpower particularly auxiliary nurse midwife (ANM) - Lack of rationale deployment of ANM - Lack of flexibility in the programme to allow rationale adjustments in AVD payments - Lack of awareness of the community about immunisation - Fear of AEFI amongst caregivers |
| 81 | Derso et al., 2020 [94] | Ethiopia, Africa | L | Sub- national | 2019 | Cross- sectional | RV | Demand- side | Enablers:   - Maternal education: formal education - Travel time to vaccination centers: less than 1 hour - Postnatal care |
| 82 | Murakami et al., 2008 [35] | Vietnam, Asia | LM | National | 2004 | Mixed- methods | Hep-B BD | Supply- and demand- side | Enablers:   - Pregnancy tracking by CHWs and VHWs   Barriers:   - Ineffective pregnancy tracking - Large hosptal established guidelines that sometimes do not follow national policy - Absence of good working relationship between private practitioners and EPI - Newborns weighing less than 2500 g or sick on the day of birth - Fear of neonatal deaths or adverse events following birth dose; that would be linked by mothers to the vaccine - Traditional practice of sequestering newborns at home during first month after delivery - Grandmother disagreed with birth dosing since no obvious benefit for healthy-looking newborn |
| 83 | Gupta et al., 2025 [70] | India, Asia | LM | National | 2019- 2021 | Cross- sectional | Hep-B BD | Demand- side | Barriers:   - Place of residence: rural - Religion: Christians - Mothers' education: lower-primary or no formal education - Media exposure: No - Birth order: four and above |
| 84 | Parida et al., 2025 [89] | India, Asia | LM | Facility | 2019- 2020 | Cross- sectional | BCG, OPV BD, RV | Demand- side | Enablers:   - Maternal education: high secondary and above - Paternal education: high secondary and above |
| 85 | Brito et al., 2017 [138] | Domini- can Republic, Caribbean | UM | National | 2014 | Cross- sectional | BCG, Hep-B BD | Demand- side | No significant enablers and barriers were reported |
| 86 | Mansour et al., 2016 [99] | Lebanon, Asia | LM | National | 2015- 2016 | Cross- sectional | Hep-B BD | Demand- side | Enablers:   - Age of the child (months: older) - Place of last vaccination: other, hospital etc   Barriers:   - Nationality: Syrian - Main source of vaccine information: Nursery/school |
| ***Grey literature*** | | | | | | | | | |
| 87 | Suharti et al., 2013 [139] | Indonesia, Asia | UM | Facility | 2012 | Cross- sectional | Hep-B BD | Demand- side | Enablers:   - Cultural/religious belief - Family influence |
| 88 | Manurung et al., 2023 [107] | Indonesia, Asia | UM | Facility | 2022 | Cross- sectional | Hep-B BD | Supply- and demand- side | Enablers:   - Midwife’s motivation - Midwife’s skill - Midwife’s perception |
| 89 | Yuliasnita et al., 2024 [90] | Indonesia, Asia | UM | Facility | 2023 | Cross- sectional | Hep-B BD | Demand- side | Enablers:   - Caregiver’s knowledge - Caregiver’s education level |
| 90 | Rachman et al., 2015 [108] | Indonesia, Asia | UM | Facility | 2015 | Cross- sectional | Hep-B BD | Supply- and demand- side | Enablers:   - Caregiver’s knowledge - Caregiver’s attitude - Health worker’s role |
| 91 | Aprilyna et al., 2025 [112] | Indonesia, Asia | UM | Facility | 2023 | Cross- sectional | RV | Demand- side | Enablers:   - Support from husband - Support from community |
| 92 | Rambe, 2016 [113] | Indonesia, Asia | UM | Facility | 2015 | Case-  control | Hep-B BD | Supply- and demand- side | Enablers:   - Absence of cultural/religious barrier - Delivery assisted by health worker |
| 93 | Siagian et al., 2021 [43] | Indonesia, Asia | UM | Sub- national | 2019- 2020 | Cross- sectional | Hep-B BD | Supply- side | Enablers:   - Supervision to frontline worker - Working experience of frontline worker |
